# Comparative Genomics Reveals Early Emergence and Biased Spatiotemporal Distribution of SARS-CoV-2

**DOI:** 10.1093/molbev/msab049

**Published:** 2021-02-19

**Authors:** Matteo Chiara, David S Horner, Carmela Gissi, Graziano Pesole

**Affiliations:** 1 Department of Biosciences, University of Milan, Milan, Italy; 2 Institute of Biomembranes, Bioenergetics and Molecular Biotechnologies, Consiglio Nazionale delle Ricerche, Bari, Italy; 3 Department of Biosciences, Biotechnology and Biopharmaceutics, University of Bari A. Moro, Bari,Italy

**Keywords:** comparative genomics, SARS-CoV-2, evolution, early emergence, classification

## Abstract

Effective systems for the analysis of molecular data are fundamental for monitoring the spread of infectious diseases and studying pathogen evolution. The rapid identification of emerging viral strains, and/or genetic variants potentially associated with novel phenotypic features is one of the most important objectives of genomic surveillance of human pathogens and represents one of the first lines of defense for the control of their spread. During the COVID 19 pandemic, several taxonomic frameworks have been proposed for the classification of SARS-Cov-2 isolates. These systems, which are typically based on phylogenetic approaches, represent essential tools for epidemiological studies as well as contributing to the study of the origin of the outbreak. Here, we propose an alternative, reproducible, and transparent phenetic method to study changes in SARS-CoV-2 genomic diversity over time. We suggest that our approach can complement other systems and facilitate the identification of biologically relevant variants in the viral genome. To demonstrate the validity of our approach, we present comparative genomic analyses of more than 175,000 genomes. Our method delineates 22 distinct SARS-CoV-2 haplogroups, which, based on the distribution of high-frequency genetic variants, fall into four major macrohaplogroups. We highlight biased spatiotemporal distributions of SARS-CoV-2 genetic profiles and show that seven of the 22 haplogroups (and of all of the four haplogroup clusters) showed a broad geographic distribution within China by the time the outbreak was widely recognized—suggesting early emergence and widespread cryptic circulation of the virus well before its isolation in January 2020. General patterns of genomic variability are remarkably similar within all major SARS-CoV-2 haplogroups, with UTRs consistently exhibiting the greatest variability, with s2m, a conserved secondary structure element of unknown function in the 3′-UTR of the viral genome showing evidence of a functional shift. Although several polymorphic sites that are specific to one or more haplogroups were predicted to be under positive or negative selection, overall our analyses suggest that the emergence of novel types is unlikely to be driven by convergent evolution and independent fixation of advantageous substitutions, or by selection of recombined strains. In the absence of extensive clinical metadata for most available genome sequences, and in the context of extensive geographic and temporal biases in the sampling, many questions regarding the evolution and clinical characteristics of SARS-CoV-2 isolates remain open. However, our data indicate that the approach outlined here can be usefully employed in the identification of candidate SARS-CoV-2 genetic variants of clinical and epidemiological importance.

## Introduction

The ongoing Coronavirus disease 2019 (COVID-19) pandemic (Poon and Peiris 2020) poses the greatest global health and socioeconomic threat since World War II. The first case of COVID-19 was reported in Wuhan city, Hubei province, China, in late December 2019 (Zhou et al. 2020), although retrospective analyses have placed the onset as early as December 1 (Duchene et al. 2020), and other, sometimes controversial, studies indicate widespread distribution of the causal agent significantly earlier (Apolone et al. 2020; Deslandes et al. 2020; La Rosa et al. 2020).

At the time of writing, COVID-19 has affected more than 200 countries worldwide, with more than 65 million confirmed individual infections and a death toll in excess of 1.5 million. Varying criteria for reporting COVID-19-related deaths, the fact that very mild or asymptomatic infections can often go undetected, differences in testing strategies, and other demographic factors (Dowd et al. 2020; Lavezzo et al. 2020; Niedzwiedz et al. 2020), suggest that both these figures are likely to represent substantial underestimates of its worldwide impact. The first complete genome sequences of the viral pathogen were determined in early January 2020 by second generation metatranscriptomic sequencing (Wu et al. 2020), allowing the rapid development of diagnostic tests (Corman et al. 2020) and the development of molecular monitoring strategies (Qiang et al. 2020).

The viral genome is approximately 30.000 nt in size and shows high similarity (∼79%) with SARS-CoV-1 (Lu et al. 2020), a beta-coronavirus of the subgenus Sarbecovirus, and the causal agent of a large scale epidemic of viral pneumonia (Severe Acute Respiratory Syndrome, SARS) that affected China and other 25 countries in 2003 and 2004 (Vijayanand et al. 2004). The International Committee on Taxonomy of Viruses (ICTV) designated the novel pathogen SARS-CoV-2. Phylogenetic analyses have assigned SARS-CoV-2 to the Severe acute respiratory syndrome-related coronavirus (SARSr-CoV) group, where it forms a relatively distant sister group to SARS-CoV-1, interleaved with various SARSr-CoVs isolated from nonhuman mammalian species (Coronaviridae Study Group of the International Committee on Taxonomy of Viruses 2020).

SARS-CoV-2 shows the highest levels of genome identity (96%) with a bat SARSr-CoV denoted RaTG13, which was isolated in the Yunnan province in 2013 (Zhou et al. 2020). Despite this sequence similarity, SARS-CoV-2 differs from RaTG13 in several key features. Arguably, the most important of these is the presence of a polybasic furin cleavage site insertion (residues PRRA) at the junction of the S1 and S2 subunits of the Spike protein (Coutard et al. 2020). This insertion, which may increase the infectivity of the virus, is not present in related beta-coronaviruses, although similar polybasic insertions are observed in other human coronaviruses, including HCoV-HKU1, as well as in highly pathogenic strains of avian influenza virus (Nao et al. 2017). Additionally, the RBD (recognition binding domain) of the SARS-CoV-2 spike protein is significantly more similar (97% identity) to that of SARSr-CoVs isolated from specimens of Malayan pangolins (*Manis javanica*) illegally imported into southern China (Guangdong and Guangxi provinces) than to the RDB of RaTG13 (89% identity) suggesting that recombination in pangolins or other mammalian “intermediate” or “amplifying” hosts may have predated human transmission (Lam et al. 2020; Wong et al. 2020).

As the COVID-19 pandemic has progressed, the number of viral isolates for which genomic sequences are available has increased substantially and in excess of 175.000 viral genomes are currently publicly available (EpiCoV Data Curation Team 2020). As expected, considering its recent emergence and the reportedly low mutation rates of coronaviruses in general (Sanjuán et al. 2010), SARS-CoV-2 genomes show low levels of genetic diversity (average pairwise identity of 99.99%). These considerations notwithstanding, phylogenetically distinct groups of SARS-CoV-2 isolates have been identified (Benvenuto et al. 2020; Ceraolo and Giorgi 2020; Forster et al. 2020; Gudbjartsson et al. 2020; Phan 2020; Walker et al. 2020) and several of these clusters show highly biased geographic distributions. Many independent studies have tentatively linked particular genomic signatures to increased/decreased virulence or possible adaptation to human hosts (Grubaugh et al. 2020; Korber et al. 2020; Pachetti et al. 2020). Although, in the absence of careful validation, it is impossible to determine whether emerging SARS-CoV-2 genetic variants are of physiological relevance and indeed reflect adaptive evolution rather than being neutral characters derived from genetic drift and founder effects, the importance of establishing simple and reproducible systems for the delineation of genetic diversity in human pathogens is widely acknowledged (Deng et al. 2016; Armstrong et al. 2019).

Currently, the GISAID (Shu and McCauley 2017) and Netxstrain portals (Hadfield et al. 2018), represent the most comprehensive repositories of SARS-CoV-2 genome sequences, and thus constitute reference points for molecular epidemiological, population genetics, and comparative genomic studies of the novel pathogen. Both systems provide tools for comparative and phylogenetic analyses, as well as instruments dedicated to genome and variant annotation. Additionally, Nexstrain provides resources for temporo-spatial graphical data representation. Other dedicated computational infrastructures, including the EBI COVID-19 Data Portal (https://www.covid19dataportal.org/) and the SARS-CoV-2 resource portal (https://www.ncbi.nlm.nih.gov/sars-cov-2/), also facilitate access and retrieval of COVID-19-related data including raw sequences reads. Although all these platforms constitute invaluable resources for the SARS-CoV-2 research community, the exponential expansion of the viral population during the current pandemic, as well as notable temporo-geographic biases in sampling, low-sequence variability, recombination (de Wit et al. 2016; Boni et al. 2020; Phan 2020), and limited metadata associated with many sequences pose significant challenges for the rapid and effective identification of relevant genomic variants and viral haplotypes and might potentially hinder pure phylogenetics approaches. Additionally, although molecular phylogenetic methods are clearly the most suitable approach for viral transmission tracing from genomic sequences, they can be computationally burdensome and the optimization of substitution models as well as the interpretation of complex topologies imply a requirement for a certain level of technical expertise. Moreover, widespread variation in allele frequencies is normally observed during an outbreak and requires ad hoc analyses to identify minimum levels of divergence required to delineate different clusters of genomic sequences while avoiding excessive fragmentation.

Currently, the approach proposed by Rambaut et al. (2020) is regarded as the most evolutionarily accurate and consistent available method for nomenclature and classification of emerging SARS-CoV-2 viral strains. This framework applies rigorous phylogenetic analyses, combined with simple rules—based on prevalence and phylogeographic distribution—to identify novel groups or viral lineages and their ancestry and can efficiently accommodate novel sequences as they are generated. However, it is not specifically designed to identify/pinpoint highly prevalent genetic variants or rapidly emerging viral isolates, as reflected by the fact that the system currently identifies more than 230 distinct lineages which show a median size of 22.5 genomes.

In the light of the above considerations, we and others (Chiara et al. 2020; Yang et al. 2020) have proposed phenetic clustering for the delineation of current and emerging viral genetic diversity considering only genetic variants that are highly prevalent (AF > 1%) in the viral population. These methods are conceptually more simple than those based on true phylogenetic analyses, and have the advantage of providing a more general picture of genetic variants and haplotypes. However, allele frequencies are typically estimated from all sequences available at the time of study, implying the potential loss of information regarding variation in frequencies of even clinically or statistically relevant variants over time. This consideration is further complicated by extensive geographic biases in sequence sampling. In an attempt to partially address these considerations, here, we extend a simple framework inspired by multilocus strain typing (MLST), a classification approach often used for microbes (Maiden 2006) to the geo-spatial analysis of SARS-CoV-2 genomic sequences. Thus, we estimate changes in “fixed” viral allele and haplotype frequencies in geographic contexts across the time-course of the pandemic.

To demonstrate the validity of our approach, we have studied more than 175,000 complete SARS-CoV-2 genomes and derive intriguing observations regarding the origin (and by inference the timing) of the emergence of pandemic strains as well as the evolutionary mechanisms associated with the emergence of novel variants.

## Results

### Genomic Features and Evolutionary Dynamics of SARS-CoV-2

We retrieved 178,191 SARS-CoV-2 genomic sequences labeled as high coverage and putatively complete, derived from 121 countries in five continents from the GISAID EpiCoV portal (as available on November 10, 2020). We observed that a considerable number (49,260) of these reportedly complete genomic assemblies presented incomplete 3′- or 5′-UTRs (median size 29743 nts; reference genome size of 29,903 nts, including a polyA tail of 33 nts). Additionally, 25,865 genomes contained a large number of gaps and/or uncalled bases—ranging from 151 to 3,652. Stringent criteria were used to retain only sequences which were more likely to represent a nearly complete assembly of the SARS-CoV-2 genome (more than 29,850 nts in size) and containing only a limited number of uncertain bases (less than 150 Ns). A total of 102,951 genomes were therefore selected as the “high-quality set” (supplementary table S1, Supplementary Material online).

Analysis of raw genetic distances between these SARS-CoV-2 genomes (see Materials and Methods) revealed a mean of 0.535 variant sites between pairs of most similar sequences, slightly lower than the equivalent figure for late-phase isolates of SARS-CoV-1 from the SARS 2003-2004 epidemics (0.78 polymorphic sites) (Song et al. 2005). Furthermore, 63,869 (62.53%) of the high-quality SARS-CoV-2 genomes analyzed, have a perfect sequence identity (zero polymorphic sites) with respect to at least one other genome. Mutation rates were estimated according to the formula described in Zhao et al. 2004 and, consistent with the observed pattern of genetic distances, were marginally lower for SARS-CoV-2 (1.89 ± 0.53 x 10^−3^ substitutions per site per year) than for SARS-CoV-1 (2.38 ± 0.47 x 10^−3^ substitutions per site per year). Although these values are in line with those reported by other studies (Boni et al. 2020), we note that estimates of viral evolutionary rates can vary considerably with the timescale of measurement, owing to exponential population growth and/or varying selective pressures. Indeed, when more distantly related species were used to calibrate the estimation of evolutionary rates, substantially lower estimates were obtained (Boni et al. 2020).

A total of 28,222 distinct variable sites were observed among 102,951 high-quality genomes included in these analyses (supplementary table S2, Supplementary Material online). Considering the date of sampling as reported for each isolate, only 818 (2,89%) of these reached an allele frequency above 1%, the threshold above which a variant is considered fixed in a natural population (Wong et al. 2003) for at least one day. Strikingly, the majority of these variants remain highly frequent only for a limited period of time (supplementary fig. 1*A*, Supplementary Material online, median 15 days, upper quartile 35 days) possibly indicating a strong effect of drift and/or sampling biases. Notably, and consistent with this hypothesis a significant positive correlation is observed between the total time of circulation (that is the total number of days in which a variant is observed at over 1% frequency among sampled genomes) and maximum allele frequency reached (supplementary fig. 1*B*, Supplementary Material online). Interestingly, according to our analyses only 182 high-frequency variants (0.644%) (supplementary table S3, Supplementary Material online) achieve an allele frequency greater than 1% for more than 50 days in total. Importantly, when the entire collection of 178,191 SARS-CoV-2 genomes is considered, the total number of variant sites is significantly increased (38581 sites, supplementary table S2, Supplementary Material online), but the number and type of high (≥1%) frequency variant sites remains relatively constant (811 in total), suggesting a robust estimate of comprehensive allele frequencies for these sites. Of the seven sites that show a reduced prevalence, five are located within 200 nt of the 3′-terminal and two within the first 52 nt of the 5′-end of the genome, suggesting that their apparent reduction in frequency might be associated with the incompleteness of some genome sequences.

### Inference of Natural Selection Acting on Coding Regions

The MEME and FEL methods (Kosakovsky-Pond et al. 2020) were applied to the concatenated alignments of protein-coding genes of the 102,951 high-quality complete genomes to identify signals of adaptive evolution. A total of 849 sites were associated (*P* value ≤0.05) with signatures of selection according to both methods (supplementary table S4, Supplementary Material online). Of these, 398 were associated with positive selection, whereas 451 sites were deemed to be under negative selection.

A highly significant over-representation of both sites under negative and positive selection is observed among the 818 high-frequency polymorphic sites of protein-coding genes. In particular, 36 of these sites (OR = 2.97, Fisher *P* value 6.26e-08) are highlighted as evolving under positive selection, whereas 61 sites (OR = 4.44, Fisher *P* value 2.20e-16) were predicted to be under negative selection. Strikingly, this pattern is even more pronounced, when only the 182 sites that display an allele frequency of 1% or greater for 50 or more days are considered, with 17 (Fisher *P* value 5.45e-09, OR = 6.62) and 30 (Fisher *P* value 1.20e-21, OR = 10.31) sites respectively, predicted to be under positive or negative selection.

### Clustering of High-Frequency Haplotypes to Explore SARS-CoV-2 Genomic Diversity

We and others (Chiara et al. 2020; Yang et al. 2020) have proposed conceptually simple systems to monitor SARS-CoV-2 genetic diversity and complement phylogenetic methods. Here, we develop this philosophy, employing an approach inspired by MLST (Maiden 2006), where only variants that reach a relatively high prevalence (typically 1%, a frequency that is often considered to represent fixation; Wong et al. 2003) are considered and clustering of allele presence/absence profiles identifies recurrent viral haplotypes.

In the context of the extreme variation between allele frequencies observed for SARS-CoV-2, exclusion of low-frequency variants, which, as previously demonstrated, typically show short temporal persistence, is potentially helpful in the search to capture significant trends in variation of genomic diversity.

To accommodate these considerations, we propose a set of arbitrary, but empirically reasonable conditions for the operational classification of SARS-CoV-2 haplotypes:

1. Given that closely related genomes show an average of 0.535 polymorphic sites, we suggest that distinct haplogroups (HGs) should differ by least two high-frequency polymorphic sites.
2. To avoid an excessive fragmentation, each haplogroup should incorporate at least 100 distinct genomes.
3. Since the variability of SARS-CoV-2 is limited, haplogroups that share one or more high-frequency polymorphic sites (have one or more nucleotide substitution in common) should form “macrohaplogroups” (MHGs). Genetic markers defining macrohaplogroups should be “completely” fixed (i.e., AF >0.9) in the associated haplogroups.
4. To minimize the effects of sampling biases, analyses of allele frequencies should be performed for distinct time windows over the course of the COVID-19 pandemic.
5. To mitigate the impact of short-term effects, only variants that show high frequency (above 1%) for a relatively long span of time, (arbitrarily set to a minimum of 50—not necessarily consecutive—days in this study) should be included in the final analyses.

To facilitate the classification of newly sequenced SARS-CoV-2 genomes, an automated software package that implements the criteria devised in the present study is made publicly available as a standalone tool at https://github.com/matteo14c/assign_CL_SARS-CoV-2 and, through a dedicated galaxy web-server at http://corgat.cloud.ba.infn.it/galaxy.

**Fig. 1.**
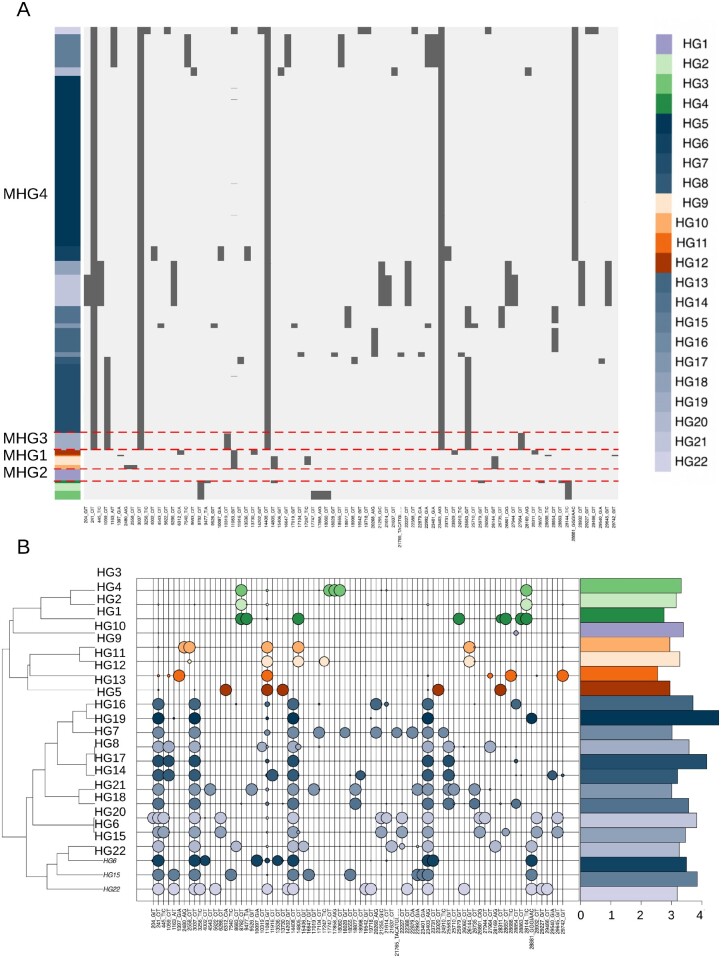
Haplogroups of 102,951 SARS-CoV-2 genomes. (*A*) Heatmap of presence/absence of 82 high-frequency polymorphic sites (AF >0.01) in 102,951 “high-quality” complete SARS-CoV-2 genomes, assigned to the 22 clusters identified in the current study. Genomic coordinates are represented on the *x* axis. Light gray indicates a reference allele, dark gray an alternative allele for that site. The panels on the left indicate haplogroups, with a different color assigned to each haplogroup. Dotted lines delineate macrohaplogroups. (*B*) Bubbleplot of allele frequency of the 82 high-frequency polymorphic sites in individual haplogroups color codes corresponding to those used in figure 1*A*. The dendrogram on the left indicates haplogroups with similar allele frequency profiles. The size of each “bubble” is proportional to the frequency of that allele in a given cluster. Barplot on the right panel indicates the number of genomes assigned to every haplogroup, scaled by logarithm base 10.

The aforementioned criteria were applied to both the 102,951 high-quality genomes (high-quality set), and the entire collection of 178,191 genomes (extended set). For the latter, terminal regions of the genome were excluded owing to the observed degree of genome incompleteness. For both data sets (fig. 1*A* and supplementary fig. S2*A*, Supplementary Material online), 22 distinct haplogroups (HG1-HG22) and four larger macrohaplogroups (MHG1-MHG4) (table 2 and supplementary table S1, Supplementary Material online) were recovered. A total of 82 (of 182) distinct high-frequency genetic variants, were “completely” fixed (relative allele frequency >0.9) in one or more haplogroups (table 1). Figure 1*B* (and supplementary fig. S2*B*, Supplementary Material online) shows that each haplogroup is defined by a characteristic molecular signature consisting between 2 and 15 high-frequency alleles (supplementary table S5, Supplementary Material online). HG1 is the only exception in this respect, as it is composed of genomes that are highly similar to the reference.

**Table 1.** List of 82 High-Frequency Polymorphic Sites.

POSa	REFb	ALTc	Selectiond	Annotatione	MAX All Freq (%)f
204	G	T	Not Applicable	5′-UTR: nc.G204T	61.03
241	C	T	Not Applicable	5′-UTR: nc.C241T	100
445	T	C	NO	nsp1: c.180T>C, p.V60V, synonymous	67.0
1059	C	T	Positive	nsp2: c.254C>T, p.T85I, missense	31.7
1163	A	T	NO	nsp2: c.358A>T, p.I120F, missense	53.2
1397	G	A	NO	nsp2: c.592G>A, p.V198I, missense	6.8
2480	G	A	NO	nsp2: c.1675A>G, p.I559V, missense	3.3
2558	C	T	NO	nsp2: c.1753C>T, p.P585S, missense	3.5
3037	C	T	Negative	nsp3: c.318C>T, p.F106F, synonymous	100.0
3256	T	C	NO	nsp3: c.537T>C, p.N179N, synonymous	7.0
4002	C	T	Positive	nsp3: c.1283C>T, p.T428I, missense	4.5
4543	C	T	NO	nsp3: c.1824C>T, p.T608T, synonymous	7.1
5622	C	T	NO	nsp3: c.2903C>T, p.P968L, missense	7.1
6286	C	T	NO	nsp3: c.3567C>T, p.T1189T, synonymous	66.2
6312	C	A	Positive	nsp3: c.3593C>A, p.T1198K, missense	2.8
7540	C	T	NO	nsp3: c.4821T>C, p.T1607T, synonymous	51.9
8683	C	T	NO	nsp4: c.129C>T, p.I43I, synonymous	7.7
8787	C	T	Negative	nsp4: c.228C>T, p.S76S, synonymous	36.1
9477	T	A	NO	nsp4: c.923T>A, p.F308Y, missense	4.2
9526	G	T	Negative	nsp4: c.972G>T, p.M324I, missense	7.1
10097	G	A	NO	nsp5: c.43G>A, p.G15S, missense	6.7
10319	C	T	NO	nsp5: c.265C>T, p.L89F, missense	6.6
11083	G	T	NO	nsp6: c.111G>T, p.L37F, missense	66.5
11916	C	T	NO	nsp7: c.74C>T, p.S25L, missense	5.1
13536	C	T	Negative	nsp12: c.96C>T, p.Y32Y, synonymous	4.5
13730	C	T	Positive	nsp12: c.290C>T, p.A97V, missense	3.0
14202	G	T	NO	nsp12: c.762G>T, p.E254D, missense	7.1
14408	C	T	Positive	nsp12: c.968C>T, p.P323L, missense	100
14805	C	T	Negative	nsp12: c.1365C>T, p.Y455Y, synonymous	27.6
15406	G	T	NO	nsp12: c.1966G>T, p.A656S, missense	7.8
16647	G	T	NO	nsp13: c.411G>T, p.T137T, synonymous	51.9
17109	G	T	NO	nsp13: c.783G>T, p.E261D, missense	7.1
17104	C	T	Positive	nsp13: c.868C>T, p.H290Y, missense	4.4
17247	C	T	Negative	nsp13: c.1011T>C, p.R337R, synonymous	3.0
17747	C	T	Positive	nsp13: c.1511C>T, p.P504L, missense	12.0
17858	A	G	NO	nsp13: c.1622A>G, p.Y541C, missense	12.4
18060	C	T	Negative	nsp14: c.21C>T, p.L7L, synonymous	12.4
18028	G	T	NO	nsp13: c.1792G>T, p.A598S, missense	3.34
18555	C	T	NO	nsp14: c.516C>T, p.D172D, synonymous	51.9
18877	C	T	Negative	nsp14: c.838C>T, p.L280L, synonymous	25.0
18898	C	T	NO	nsp14: c.959C>T, p.A320V, missense	1.3
19542	G	T	NO	nsp14: c.1503G>T, p.M501I, missense	7.1
19718	C	T	NO	nsp15: c.98C>T, p.T33I, missense	10.5
20268	A	G	Negative	nsp15: c.648A>G, p.L216L, synonymous	8.9
21255	G	C	NO	nsp16: c.597G>C, p.A199A, synonymous	66.6
21614	C	T	Positive	spike: c.52C>T, p.L18F, missense	34.7
21637	C	T	NO	spike: c.75C>T, p.P25P, synonymous	8.5
21765	TACATG	Del	NO	spike: c.203TACATG>……,p.IHV68I, inframeDel;	3.23
22227	C	T	Positive	spike: c.665C>T, p.A222V, missense	66.4
22388	C	T	NO	spike: c.826C>T, p.L276L, synonymous	7.2
22879	C	A	NO	spike: c.1317C>A, p.N439K, missense	4.3
22992	G	A	Positive	spike: c.1430G>A, p.S477N, missense	51.9
23401	G	A	NO	spike: c.1839G>A, p.Q613Q, synonymous	51.9
23403	G	A	Positive	spike: c.1841A>G, p.D614G, missense	100
23731	C	T	Negative	spike: c.2169C>T, p.T723T, synonymous	6.7
23929	C	T	Negative	spike: c.2367C>T, p.Y789Y, synonymous	2.7
24910	T	C	NO	spike: c.3348T>C, p.T1116T, synonymous;	3.32
25563	G	T	NO	orf3A: c.171G>T, p.Q57H, missense	38.5
25710	C	T	NO	orf3A: c.318C>T, p.L106L, synonymous	7.1
25979	G	T	NO	orf3A: c.587G>T, p.G196V, missense	4.0
26060	C	T	NO	orf3A: c.668C>T, p.T223I, missense	10.5
26144	G	T	NO	orf3A: c.752G>T, p.G251V, missense	30.7
26735	C	T	Negative	geneM: c.213C>T, p.Y71Y, synonymous	25.0
26801	C	G	Negative	geneM: c.279C>G, p.L93L, synonymous	66.4
27944	C	T	NO	orf8: c.51C>T, p.H17H, synonymous	46.5
27964	C	T	NO	orf8: c.71C>T, p.S24L, missense	10.3
28169	A	G	NO	orf8: c.276A>G, p.E92E, synonymous	8.5
28311	C	T	Positive	geneN: c.38C>T, p.P13L, missense	2.9
28657	C	T	NO	geneN: c.384C>T, p.D128D, synonymous	4.2
28688	T	C	Negative	geneN: c.415T>C, p.L139L, synonymous	6.0
28854	C	T	NO	geneN: c.581C>T, p.S194L, missense	25.0
28863	C	T	NO	geneN: c.590C>T, p.S197L, missense	4.1
28144	T	C	NO	orf8: c.251T>C, p.L84S, missense	37.7
28881	GGG	AAC	NO	geneN: c.608GGG>AAC, p.RG203KR, missense	81.0
28932	C	T	NO	geneN: c.659C>T, p.A220V, missenseepi27: nc.C5T, NO, NO	66.4
29227	G	T	Negative	geneN: c.954G>T, p.S318S, synonymous	7.1
29446	C	T	NO	geneN: c.1193C>T, p.A398V, missense	7.1
29540	G	A	NO	None	1.3
29645	G	T	none	orf10: c.88G>T, p.V30L, missensesl4: nc.G17T, NO, NO	66.4
29742	G	T	Not Applicable	3′-UTR: s2m	6.9

a Genomic position.

b Reference allele.

c Alternative allele.

d Type of selection according to FEL and MEME, not applicable: the site is not included in a protein-coding gene.

e Functional annotation of the site.

f Maximum prevalence (proportion of genomes with the variant).

**Table 2.** List of Haplogroups and Macrohaplogroups.

Haplogroupa	Super-Haplogroupb	Emergence (presumed)c	Classificationd
HG1	MHG1	Wuhan Dec 2019	Early
HG2	MHG2	Wuhan Dec 2019	Early
HG3	MHG2	Washington, Jan 2020	Early
HG4	MHG2	Panama, Feb 2020	Middle
HG5	MHG3	Sichuan, Jan 2020	Early
HG6	MHG3	Denmark, Feb 2020	Middle
HG7	MHG3	France, Feb 2020	Middle
HG8	MHG3	New York, March 2020	Middle
HG9	MHG4	Chongqing, January 2020	Early
HG10	MHG4	England, February 2020	Middle
HG11	MHG4	Wuhan, January 2020	Early
HG12	MHG4	Singapore, February 2020	Middle
HG13	MHG3	England, Mach 2020	Middle
HG14	MHG3	Saudi Arabia, February 2020	Middle
HG15	MHG3	Australia, April 2020	Middle
HG16	MHG3	Costa Rica, March 2020	Middle
HG17	MHG3	Norway, July 2020	Late
HG18	MHG3	Netherlands, May 2020	Middle
HG19	MHG3	USA, March 2020	Late
HG20	MHG3	England, May 2020	Late
HG21	MHG3	England, July 2020	Late
HG22	MHG3	Bangladesh, June 2020	Late

a Haplogroup name.

b Macrohaplogroup.

c Place and time of isolation of the first isolated SARS-CoV-2 genome assigned to that haplogroup.

d Classification of haplogroups in early, late, and middle according to the criteria defined in the current study.

**Fig. 2.**
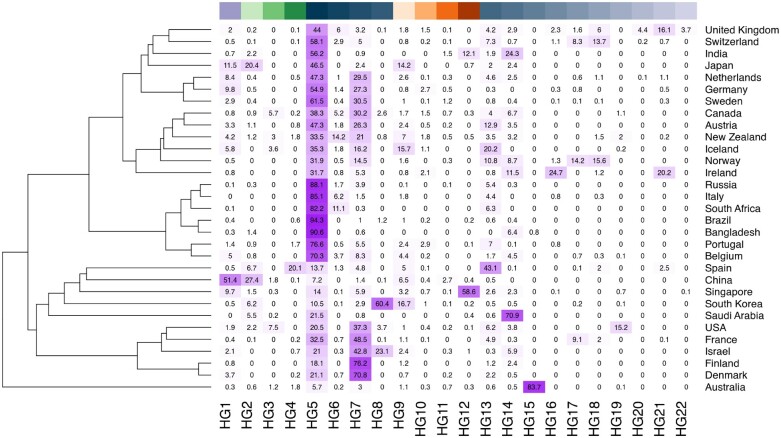
Worldwide prevalence of SARS-CoV-2 haplogroups. Heatmap of worldwide prevalence of SARS-CoV-2 haplogroups. Only countries for which at least 100 distinct genomes of SARS-CoV-2 are available in a public repository are shown. Color codes on the top indicate individual haplogroups, according to figure 1.

**Fig. 3.**
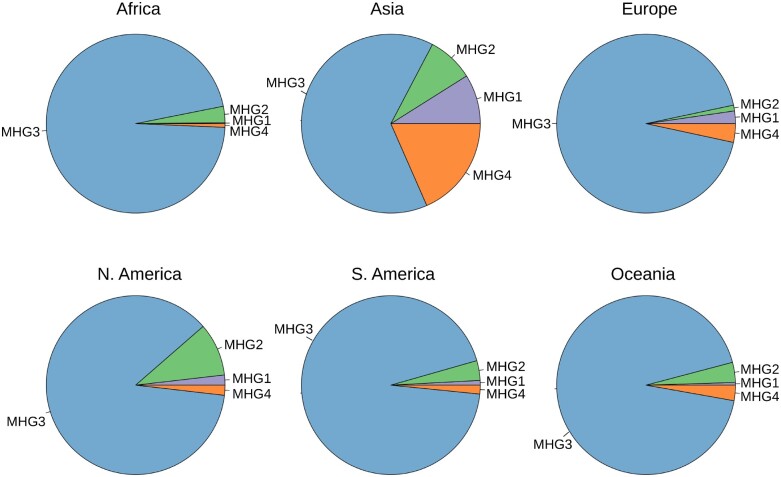
Prevalence of SARS-CoV-2 macrohaplogroups in different continents. Pie-chart of prevalence of types of SARS-CoV-2 macrohaplogroups in different continents. Color code as in figure 1.

Consistent with other reports (Benvenuto et al. 2020; Ceraolo and Giorgi 2020; Forster et al. 2020; Gudbjartsson et al. 2020; Phan 2020; Walker et al. 2020), we observe (fig. 2) a highly biased geographic distribution of SARS-CoV-2 haplogroups worldwide. Indeed, although relatively balanced proportions of each haplogroup are observed in Asia as a whole, the majority of all viral genomes observed in other continents are assigned to a single macrohaplogroup: MHG3 (fig. 3). This is also reflected by an extreme increase over time in the frequencies of the genetic variants that define the MHG3 (supplementary fig. S3, Supplementary Material online). Additionally, several haplogroups identified by our analyses have a high prevalence only in a limited number of countries (fig. 2), while are overall less prevalent worldwide. For example, HG15 is highly prevalent only in Australia, whereas conversely HG12 is highly frequent only in India and Singapore. Similarly, HG21 is observed principally in Ireland and in the United Kingdom, and HG19 seems to be specific to the United States. Consistent with these findings, patterns, and dynamics of prevalence of distinct HGs are considerably different when distinct countries/geographic locations are compared. For example (supplementary fig. S4, Supplementary Material online), the rapid emergence of HG15 in Australia coincides with a decrease in the total number of genomes sequenced, at time T0 (the collection date of the reference genome)+150 days and with a concurrent reduction of the prevalence of all other previously circulating HGs (supplementary fig. S4*A*, Supplementary Material online). Conversely in the United States, different HGs, all incorporated within MHG3, appear approximately at the same time point (T0 + 70), but become highly prevalent at different intervals in time (supplementary fig. S4, Supplementary Material online). Finally, in the UK HG5, the most prevalent haplogroup in the country for a long period of time, is now being gradually replaced by HG21 (supplementary fig. S4, Supplementary Material online). Notably, and consistent with our previous observations, a rapid decrease in the prevalence of genomes associated with MHG1, MHG2, and MHG4 is observed after time T0 + 70, whereas relatively high prevalence of these genomes were observed in many countries during the early phase of the pandemic.

Importantly, from supplementary figure S5, Supplementary Material online, we notice that there is a modest prevalence of MHG3 in China, the country that is currently considered the origin of the outbreak, where it accounts for only 5.1% of all the genomes available.

Of the 82 high-frequency polymorphic sites that reach complete, or nearly complete fixation in at least one haplogroup (table 1 and fig. 1*B*) only two variants (11083 G->T; 14805 C->T;) show an allele frequency ≥0.01 in more than one macrohaplogroup, whereas the remaining 80 have AF ≥ 0.01 in only one macrohaplogroup, and can therefore be considered MHG “specific.” These observations strongly support our contention that high-frequency variable sites, as defined here, are effective for the discrimination/classification of distinct genomic signatures in SARS-CoV-2.

Strikingly, 24 of the 77 sites associated with protein-coding genes that are fixed in and specific to at least one haplogroup are predicted to be under positive (9) or negative (14) selection according to FEL or MEME (table 1). Although this observation might be suggestive of distinct phenotypic features/properties for the different SARS-CoV-2 types, as previously suggested by other authors (Grubaugh et al. 2020; Korber et al. 2020; Pachetti et al. 2020), in the absence of experimental validation, such inferences should be treated carefully.

### Spatiotemporal Distribution of SARS-CoV-2 Genome Types and Emergence of New Types

Although phylogeographic analyses show a highly biased distribution of SARS-CoV-2 genomes worldwide (figs. 2 and 3; table 2), representatives of each macrohaplogroup are already observed in different geographic regions of China—the presumed country of origin of the outbreak—within 25 days of the report of the first case of COVID19 in Wuhan (supplementary fig. S5*A*, Supplementary Material online). During the same time period, three distinct haplogroups belonging to a different macrohaplogroup (MHG1, MHG2, and MHG4), were already observed in Wuhan (supplementary fig. S5*B*, Supplementary Material online), whereas genomes from MHG3, carrying the 23403 A > G substitution (causing the D614G spike variant) are observed both in Guangdong and Zhejiang in this initial phase.

**Fig. 4.**
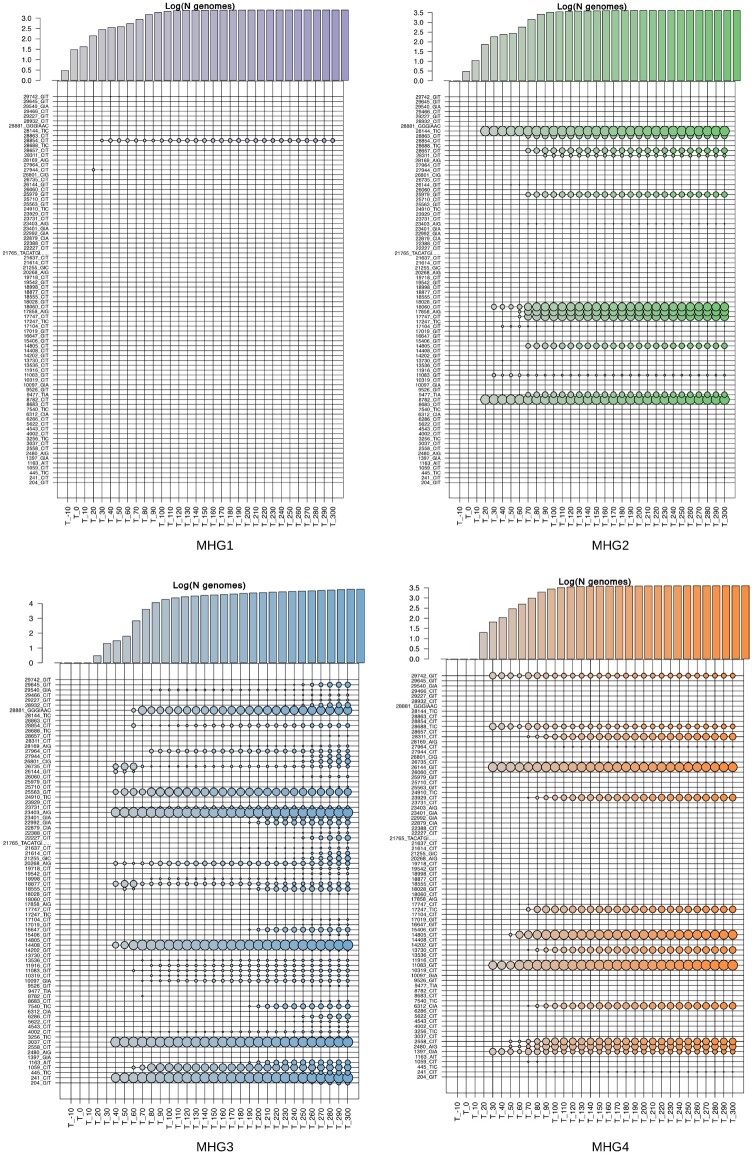
Frequency of highly prevalent alleles in SARS-CoV-2 macrohaplogroups at different time intervals. Bubbleplots of allele frequency in the four macrohaplogroups of SARS-CoV-2 genomes, at different intervals in time. Each bubbleplot displays the allele frequency of the 82 high-frequency polymorphic sites calculated at different, nonoverlapping, intervals of 10 days. (“T_” with time 0 = December 26, 2019). The size of each bubble is proportional to the allele frequency. Color codes according to haplogroups as in figure 1. The barplots above indicate the number of genomes of each macrohaplogroup observed at each time interval considered, scaled by logarithm base 10.

Strikingly, we notice that, among the 82 high-frequency variants, that define the major haplogroups of SARS-CoV-2, 28 are also present in one or more genomes of SARSr-CoV-2 isolated from bat and/or pangolin specimens (supplementary fig. S6, Supplementary Material online). These alleles appear to be highly admixed among SARSr-CoV-2 coronaviruses isolated from pangolins and bats, suggesting possible parallelism/convergence, but potentially suggestive of extensive recombination between immediate ancestors of SARS-CoV-2. To investigate possible scenarios of emergence of novel genome types, the allele frequency distribution of the 82 genetic variants that define the major haplogroups of SARS-CoV-2 (AF ≥ 0.01) were compared at intervals of 10 days since December 26, 2019 (the collection date of the reference genome). Within haplogroups, distributions of allele frequency are highly stable and do not change over time (supplementary figs. S7–S9, Supplementary Material online). Since by definition major SARS-CoV-2 viral haplotypes identified in this study are formed by at least two or more characteristic genomic variants, this suggests that the majority of the genomic signatures that define distinct haplogroups concomitantly reached a high allelic frequency. This is even more evident when allele frequency distributions are compared within macrohaplogroups, as several clusters of alleles show a rapid emergence and almost immediate fixation (fig. 4 and supplementary tables S6 and S7, Supplementary Material online). Notably, we observe that genetic variants associated with HG18 and HG21, are rapidly becoming more prevalent. Importantly, we notice that both groups incorporate emerging variants that alter the sequence of the spike protein (supplementary fig. S10*A* and *B*, Supplementary Material online): L18F (HG21), and A222V (both HG18 and HG21). Both variants are predicted to be under positive selection according to FEL and MEME (table 1). The rapid emergence of the A222V variant, which probably originated in Spain, has already been described in (Hodcroft et al. 2020). Although it might be tempting to speculate that, similar to the D614G variant in the spike protein—the hallmark of MHG3—these rapidly emerging variants might be associated with increased viral fitness, other nonsynonymous spike protein variants, such as S477N in HG15, also show signatures of positive selection and apparent increase in allele frequency, but subsequently exhibit rapid decrease in prevalence (supplementary fig. S10*C*, Supplementary Material online).

In addition to rapid selection of standing variation as an adaptive process (Nowak and Schuster,1989), other evolutionary processes, including genetic drift and founder effects, can explain rapid changes in allele frequency (Holland et al. 1991; Lynch et al. 2016). Indeed, the proposed importance of “superspreader” events and individuals and the inferred overdispersion of *R*_0_ associated with SARS-CoV-2 transmission patterns (e.g., Endo et al. 2020; Gómez-Carballa et al. 2020) might be consistent with an important role for founder effects, particularly in the context of containment strategies imposed in many countries after the initial outbreak and after the initiation of “second waves.”

**Fig. 5.**
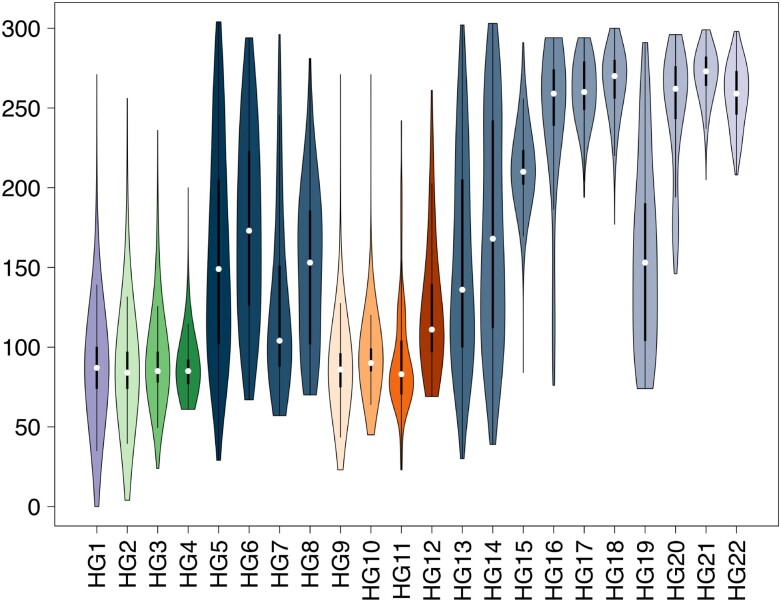
Times of emergence and circulation of SARS-CoV-2 haplogroups Violin plots of isolation dates of SARS-CoV-2 strains assigned to each haplogroup of SARS-CoV-2 genomes. Color codes according to figure 1. Haplogroups are indicated on the *x* axis. Isolation dates are reported on the *y* axis.

Comparison of isolation dates (fig. 5 and supplementary fig. S11, Supplementary Material online), suggest that the majority of currently observed haplogroups of SARS-CoV-2 were not present during the initial phases of the pandemic and seem to emerge at a later time. Using arbitrary thresholds, based on days of first isolation, haplogroups can be roughly divided into “early” (HG1-HG3, HG5, HG9, HG11 appearing within 30 days of the isolation of the reference genome), “middle” (HG4, HG6-HG8, HG10, HG12-HG15, and HG19: appearing between 30 to 100 days), and “late” (HG17, HG18, HG20-HG22: appearing after 100 days) (table 2). Consistent with our previous findings, we observe that “early” haplogroups have a probable origin in China, as shown in the comparison of the phenetic patterns and the localities of the first 50 isolates (in terms of date, see supplementary fig. S12, Supplementary Material online). Conversely, the “middle” and “late” haplogroups are likely to have become distinct outside China (supplementary figs. S13 and S14, Supplementary Material online).

**Fig. 6.**
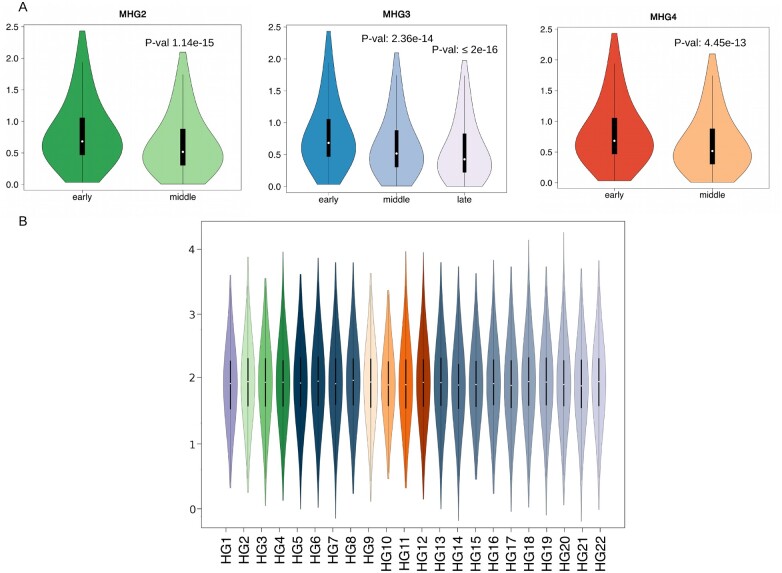
Comparison of genetic diversity of early, middle, and late haplogroups. (*A*) Violin plots of genetic diversity of early, middle, and late haplogroups of SARS-CoV-2 genomes. *P* values, for the significant reduction of genetic diversity (reduced number of distinct polymorphic sites per genome) are reported on the top of each violin plot. (*B*) Violin plot of substitution rates of the 22 clusters of SARS-CoV-2 genomes identified in this study. Color codes according to figure 1.

To discriminate between possible evolutionary scenarios associated with the rapid emergence of novel haplogroups in SARS-CoV-2, we reasoned that although founder effects or selection should be associated with an overall reduction in genomic diversity of viral sub-populations, convergent evolution should not alter the underlying allele frequency distribution (except at the sites under selection). Accordingly, we compared average allele diversity between genomes associated with “early,” “middle,” and “late” emergence within each major haplogroup (fig. 6*A*). The results show a statistically significant reduction of genetic diversity for middle and late HGs (i.e., emerging after ≥30 days) compared with early haplogroups (Wilcoxon sum and rank test *P* values 1.145e-15, 2.367e-14, and 4.45e-13 for early vs. middle in HG2, HG3, and HG4 respectively; *P* value ≤2e-16 for early compared with late). Intriguingly, a moderate but still statistically significant reduction is observed also when middle and late haplogroups in MHG3 are compared (*P* value 0.000103). Importantly, (fig. 6*B*), we observe that evolutionary rates are highly homogeneous and do not show detectable changes between haplogroups, suggesting that reduced diversity of late clusters is not associated with a reduction of evolutionary rates. According to our starting hypothesis, and in the light of the biased geographic sampling and prevalence of different HGS, these results suggest that the emergence of novel SARS-CoV-2 genome types is unlikely to be driven by widespread convergent evolution and independent fixation of advantageous substitutions.

Remarkably, our analyses do not support an increased genomic diversity for haplogroups included in MHG3 compared with other MHGs, although the 14408 C > T substitution causing the P323L variant in the nsp12 gene (RdRp) was previously described as associated with an increased genomic variability (Pachetti et al. 2020). We speculate that biased/incomplete sampling of MHG3 during the early phase of the pandemic, and the fact that Pachetti et al. compared raw nonnormalized genetic distances (instead of normalized evolutionary rates) are the most likely explanation for this discrepancy.

### Distribution of Variable Sites along the SARS-Cov-2 Genome

**Fig. 7.**
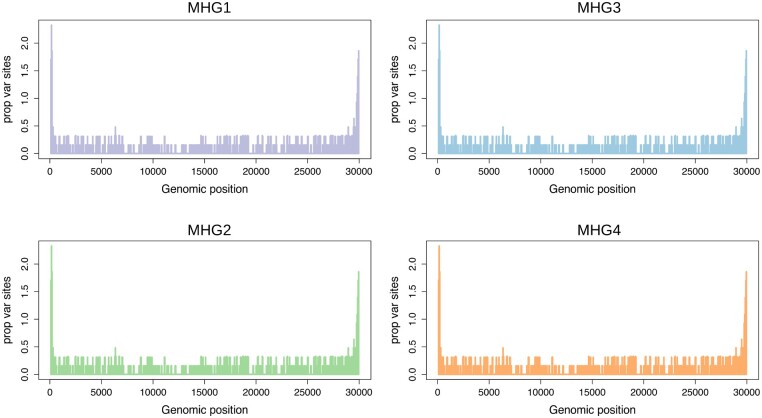
Profile of genome-wide genetic variability of SARS-CoV-2 macrohaplogroups. Plot of genomic variability—calculated as the proportion of variable sites identified in overlapping genomic windows of 100 bp—in the four macrohaplogroups MHG1-MHG4. Genomic coordinates are represented on the *x* axis, number of variable sites per window on the *y* axis.

**Fig. 8.**
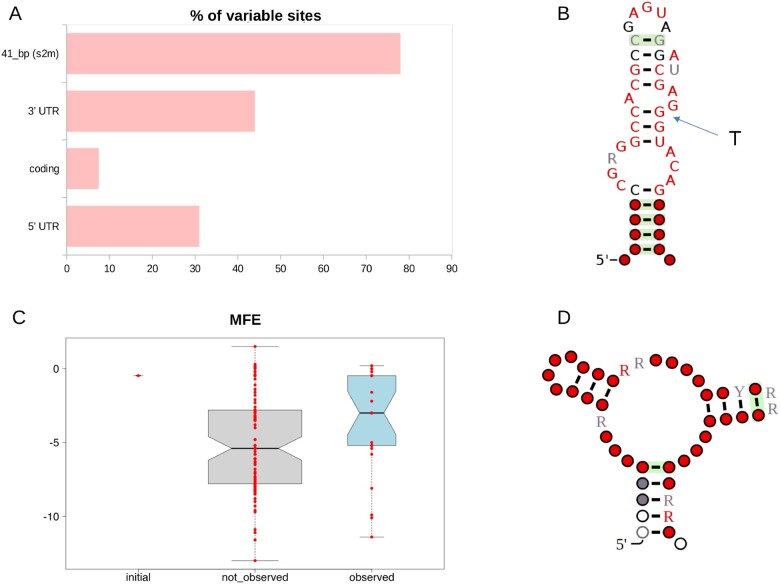
Analysis of structural stability and secondary structure of the s2m element in SARS-CoV-2. Analysis of variability and structural stability of the s2m secondary structure element. (*A*) Barplot of variability of different categories of genomic elements in the genome of SARS-CoV-2. Variability is reported as the proportion of polymorphic sites. (*B*) Consensus secondary structure of the s2m element of coronaviruses according to the RFAM model RF00164 (https://rfam.org/family/RF00164). The arrow indicates the nucleotide substitution observed in the s2m of the reference genome of SARS-CoV-2 (position 29,758). (*C*) Boxplot of MFE (minimum free energy) of predicted s2m secondary structures. Initial: MFE of the s2m element in the SARS-CoV-2 reference genome. Not observed: MFE of secondary structures associated with single nucleotide substitutions that are not observed in s2m of extant SAR-CoV-2 genomes. Observed: MFE of secondary structures associated with nucleotide substitutions found in the s2m element of extant SAR-CoV-2 genomes. (*D*) Prediction of secondary structure cofolding of s2m of SARS-CoV-2 according to the Rscape program. Color codes are used to indicate the level of conservation of single nucleotide residues according to the convention used in RFAM (white ≤50%, gray >50% and ≤75%, black >75%, and <90%, red ≥90%).

Profiles of genomic variability for each of the haplogroups and macrohaplogroups defined in this study were established using windows of 100 bp in size and sliding by 50 bp. As shown in figure 7 and supplementary figure S15, Supplementary Material online, the observed patterns are remarkably similar suggesting common patterns of variation. Density of polymorphic sites is significantly enriched (adjusted Fisher test *P* value ≤1e-15 and ≤1e-12 respectively) in both the 5′- and 3′-UTR regions, whereas protein-coding loci (CDS) show less variability. Strikingly, a single genomic region in the 3′-UTR accumulates ∼10x more mutations than any CDS, and ∼ 2x more than any other UTR region, and is the single most variable region in the genome of SARS-CoV-2 (fig. 8*A*). This highly variable genomic region is associated with a conserved secondary structure (fig. 8*A*), known as s2m (Tengs and Jonassen 2016). Strikingly, no variation is observed in s2m among 73 available SARS-CoV-1 genome sequences from the 2003-2004 epidemic or in any other currently available SARSr-CoV-2 genome.

S2m is a 43-nucleotide element that has been described in several families of single-stranded RNA viruses, including Astroviridae, Caliciviridae, Picornaviridae, and Coronaviridae (Tengs and Jonassen 2016). The molecular function of this potentially mobile structural element is not well understood. Current hypotheses include a role in the hijacking of host protein synthesis through interactions with ribosomal proteins (Robertson et al. 2005), and RNA interference (RNAi) via processing of the s2m elements into a mature microRNA (Tengs et al. 2013). In coronaviruses, the highly conserved nature s2m has also allowed the development of a PCR-based virus discovery strategy (Jonassen 2008).

As outlined in figure 8*B*, compared with the consensus secondary structure of s2m described in the Rfam database, the reference genome of SARS-CoV-2 harbors a nucleotide substitution at a highly conserved and structurally important position, with possible impacts on structural stability (the T at the SARS-CoV-2 genomic position 29,758, indicated by an arrow in fig. 8*A*). Secondary structure predictions suggest that of all possible 129 single nucleotide substitutions in the presumably ancestral sequence of s2m, as observed in the genome of RaTG13 SARSr-CoV-2, this would be the second most destabilizing in terms of minimum free energy (MFE) (supplementary table S8, Supplementary Material online). Based on this observation and on the high levels of variation of the entire s2m region, it is tempting to speculate that s2m could either be subject to diversifying selection in SARS-CoV-2, or have lost significant purifying constraints. Strikingly, we observe that the G->T substitution at 29,742, which is a hallmark of haplogroup 11, would result in a substantially increased stability of s2m (supplementary table S7, Supplementary Material online), with an MFE that becomes substantially lower than that of the s2m structure of the reference genome. Interestingly, we observe that this variant also achieves a relatively high frequency (max 6.4%) also in haplogroup 8, a possible hint of convergent evolution.

Conversely, five other recurrent substitutions in s2m, that achieve an allele frequency of 1% during the time course of the SARS-CoV-2 pandemic (29,742 G > A and 29,734 G > T, 29736 G > T, 29751 G > T and 29747 G > T) are not associated with a specific haplogroup and are predicted to result only in a marginal decrease of the MFE of the s2m secondary structure (supplementary table S8, Supplementary Material online). Interestingly, we notice (fig. 8*C*) that the same consideration applies to the majority of the nucleotide substitutions that are observed in the SARS-CoV-2 s2m element. Indeed, with respect to the background of all possible nucleotide substitutions that could occur in s2m of the SARS-CoV-2 reference genome, the set of variants that is actually observed in extant SARS-CoV-2 genomes are associated with only a modest increase in the thermodynamic stability of the structure.

A cofolding analysis of all distinct variants of the s2m elements found in the 102,951 complete and high-quality genomes—according to the criteria defined in this study—suggests a very degenerate secondary structure of s2m in SARS-CoV-2 (fig. 8*D*).

Notably although a substitution that restores the presumably ancestral state of s2m (i.e., the secondary structure of RaTG13 SARSr-CoV-2) is observed (29758 T > G), this substitution is associated only with a very limited number of genomes (103, AF = 0,00100841).

## Discussion

Effective approaches for the interpretation of genome sequences are fundamental for monitoring and understanding the dynamics of the spread and evolution of pathogens, and the SARS-CoV-2 paradigm, given both its global significance and the availability of modern sequencing technologies is particularly instructive in this sense.

In the current study, we propose a rational and reproducible approach for the delineation of genomic diversity in SARS-CoV-2 which also takes into account the temporal distribution of allele frequency by building on an informative set of variable sites, which show high prevalence in the viral population for a relevant frame of time. Applying our system to the entire collection of (more than 175,000) genomic sequences, as available on November 10, 2020, we derive interesting observations concerning evolutionary patterns of SARS-CoV-2.

We observe a low level of variability and infer a relatively low mutation rate (1.84 sites per 10^−3 ^nt per year) in SARS-CoV-2 which is consistent with previous estimates (Zhao et al. 2004; Sanjuán et al. 2010). The presence of representatives of different viral haplogroups during the early phases of the pandemic (within 25 days of the report of the first case of COVID19 in Wuhan) in several distinct geographic regions of China, is suggestive of an early circulation of SARS-CoV-2 in humans, probably well before the major outbreak of COVID19 in Wuhan. These observations, coupled with evidence provided by several other studies, would indicate an early spillover of SARS-CoV-2 to humans (Apolone et al. 2020; Deslandes et al. 2020; La Rosa et al. 2020; Zehender et al. 2020). Careful monitoring of the evolutionary rates of SARS-CoV-2 over a longer period of time, and ideally also on an unbiased/matched number of genomes isolated from different geographic areas, are required to confirm these inferences.

In this respect, the fact that a relevant number of SARS-CoV-2 high-frequency, and macrohaplogroup-specific polymorphic sites are found also in viral strains isolated from pangolins and bats specimens highlights an unexplored diversity shared by SARS-CoV-2 and SARSr-CoV-2 viral genomes. Moreover, the observed pattern of admixed SARS-CoV-2 haplogroup-specific alleles in the genomes of closely related SARSr-CoV-2 coronaviruses isolated from bat and pangolin specimens (supplementary fig. S6, Supplementary Material online), is highly consistent with possible recombination events, as suggested also by previous studies (Boni et al. 2020; Lam et al. 2020; Wong et al. 2020). In the light of these observations, it is evident that additional sampling of a substantially larger number of viral specimens, isolated from nonhuman hosts will be required to reconstruct a more complete phylogeny and to possibly trace back the “original” spillover event. Indeed, notwithstanding the high levels of similarity to SARS-CoV-2 (in the order of 97%), RaTG13, the most closely related viral genome isolated from a bat specimen, is estimated to have diverged from SARS-CoV-2 more than 25–40 years ago (Boni et al. 2020).

Our classification system, based on 82 high frequency, stable polymorphic sites, identifies a total of 22 distinct prevalent haplogroups and four macrohaplogroups of SARS-CoV-2 genome types, all having a highly biased phylogeographic distribution (figs. 2 and 3). We note that several polymorphic sites that are specifically associated (completely fixed) with haplogroups and macrohaplogroups are predicted to be either under positive or negative selection according to state of the art methods for the study of evolutionary constraints in protein-coding genes (table 1). Interestingly, several of these sites have previously been highlighted by other studies and tentatively associated with increased virulence and/or increased mutation rates of SARS-CoV-2 (Grubaugh et al. 2020; Korber et al. 2020; Pachetti et al. 2020).

Although fixation of advantageous variants has previously been proposed as an effective and widespread mechanism for the rapid increase of the fitness of a viral population (Moya et al. 2004), the functional relevance of these genomic variants remains, for now, unclear. We emphasize the importance in functional and clinical validation as reduced levels of variability, high levels of recombination, transmission dynamics, and, particularly, biased sampling of genomic sequences, might impair the accuracy of methods based on phylogenetic reconstruction of ancestral states for the identification of selective signatures (Ives et al. 2007; Som 2015). In this respect, our observation of reduced genetic variability of middle and “late” viral haplogroups belonging to (fig. 6*A*), coupled with the highly biased phylogeographic distribution of SARS-CoV-2 genome types (figs. 2 and 3), might be more consistent with genetic drift and founder effects rather than ongoing adaptive evolution. We are unaware of transmission modeling studies that have incorporated both overdispersion of the *R*_0_ transmission parameter and local distributions of observed allele frequencies, but speculate, consistently with other studies (e.g., Gómez-Carballa et al. 2020), that the impact of superspreader events and individuals, coupled with lockdowns and mobility restrictions might contribute to the rapid changes in allele frequencies observed for some variants. However, the hypothesis that drift and stochastic factors account for a great part of SARS-CoV-2 variability does not exclude selection having driven the fixation of a small number variants, so sites identified as candidates for selective evolution warrant further functional characterization both in vitro and eventually in vivo. We note that analyses of the prevalence of different HGs in different countries for which a large number of SARS-CoV-2 genomes are publicly available highlight contrasting dynamics. For example, the widespread variability in the prevalence of different HGs at different time points, which is observed in the United States might be indicative of different/distinct chains of infection or local outbreaks. Conversely, the steep reduction in the prevalence of all other HGs and the rapid emergence of HG15 in Australia, which coincides also with a substantial decrease in the number of genomes sampled at a specific time point, might be associated with a bottleneck effect caused by the implementation of containment measures and national lockdowns. Finally, the rapid increase of HG21 in the United Kingdom, a novel haplogroup that incorporates a nonsynonymous variant in the spike protein (Hodcroft et al. 2020) should be monitored carefully as the hypothesis that this mutation might represent an event of adaptive evolution can not currently be excluded.

The observation of highly divergent/geographically biased patterns of allele frequency distributions in the SARS-CoV-2, coupled with large differences in the number of genomic sequences sampled from different geographic areas or countries might represent a relevant limitation for this work. Indeed, these considerations might compromise estimation of allele frequencies, and thus limit the sensitivity of our approach in the identification of relevant/important genetic variants for which only a limited number of representative genomic sequences are available. For example, the majority of the largest HGs identified by our approach are associated with countries from which the largest number of genomes are available (UK, USA, and Australia). This suggests that currently available sampling offers only a partial overview of SARS-CoV-2 genomic variability. Importantly, approaches/nomenclature systems based on true phylogenetic analyses do not suffer from this limitation, as the delineation of distinct lineages is not determined by their overall prevalence. However, and for the same reason, results of phylogenetic analyses might be more difficult to interpret and do not facilitate the rapid identification of highly prevalent/emerging genetic variants. As such, we believe that systems for the monitoring of the evolution of SARS-CoV-2 should integrate both types of approaches and routinely incorporate geographic and temporal dimensions.

Notably, we observe a highly consistent pattern of nucleotide substitution in SARS-CoV-2 genomes between all haplogroups and macrohaplogroups, characterized by an increased variability at UTRs, in spite of the fact that a significant proportion of genomic assemblies annotated as “full-length” in GISAID are incomplete at the terminal ends. Although this incomplete representation of genomic sequences does not affect the classification system proposed in this study, it might result in an inaccurate/incomplete representation of ongoing evolution of SARS-CoV-2. This is exemplified by the s2m element, a highly conserved secondary structure element located in the 3′-UTR which carries a substitution in the reference genome of SARS-CoV-2 that destabilizes the secondary structure and is possibly functionally significant.

The s2m element shows a patchy phylogenetic distribution among distinct groups of positive-sense RNA viruses (picornaviruses, astroviruses, and coronaviruses) and likely represents a “mobile” element (Tengs and Jonassen 2016). When present it is always found in the 3′ region of such genomes (Jonassen 2008) and shows extremely high levels of conservation at the structural and primary sequence levels. However, the patchy distribution within groups of viruses with extremely similar gene complements may argue against an essential role for this element—implying that it is advantageous in specific conditions.

The substantial increase of genomic variability observed in the s2m locus, compared with the rest of the genome (as well as the observation that among 73 available SARS-CoV-1 genome sequences from the 2003-2004 epidemic and other SARSr-CoV-2 genomes, no variation is observed in s2m), suggest changes in selective pressures acting on this element. It remains unclear whether these changes reflect ongoing widespread diversifying selection in SARS-CoV-2, or whether the original disruptive substitution inactivated s2m function, leading to a general loss of significant purifying constraints. Patterns of single nucleotide substitutions in s2m provide contrasting evidence concerning the evolutionary patterns of this secondary structure element in SARS-CoV-2, as the most prevalent substitutions (29,742 G->T) seems to be associated with a considerable increase in secondary structure stability, but the majority of the substitutions observed in extant SARS-CoV-2 genomes are not optimal in terms of the recovery of a highly stable secondary structure, and, in particular, do not recapitulate the consensus sequence/structure. The functional role of s2m remains unclear: it has been proposed, on the basis of structural similarity to rRNA structural elements, that it might be involved in the regulation of translational efficiency of viral transcripts (Robertson et al. 2005). We also note that viral RNA secondary structures have been implicated in the suppression of antigen presentation (EBNA-1; Apcher et al. 2010; Tellam et al. 2012) and in the suppression of host innate immune responses (Vandevenne et al. 2010; McFadden et al. 2013; Witteveldt et al. 2014; Murat and Tellam 2015). We believe that detailed experimental evaluation of both s2m function and the possible phenotypic consequences of changes observed among SARS-CoV-2 isolates should represent a priority topic in coronavirus research.

Although, many questions concerning the mechanisms of evolution and the phenotypic characteristics of SARS-CoV-2 (increased/decreased virulence) remain open, approaches such as that presented here facilitate rapid grouping of frequent SARS-CoV-2 haplotypes and can be useful both for monitoring the changing prevalence of different types of SARS-CoV-2 and for the study of the molecular processes that underlie the emergence of novel viral types.

## Materials and Methods

A collection of 178,191 putatively complete, high-coverage SARS-CoV-2 genomes and associated metadata was retrieved from the GISAID EpicoV (Shu and McCauley 2017) platform on November 10, 2020. A total of 13 SARSr-CoV genome sequences isolated from nonhuman hosts, including bats and pangolins (Lam et al. 2020; Wong et al. 2020), were also retrieved from the GISAID EpiCoV portal at the same date. SARS-CoV-2 sequence comparisons were performed using the reference Refseq (O’Leary et al. 2016) assembly NC_045512.2, collected on December 26, 2019 and identical to the sequence of the oldest SARS-CoV-2 isolates, dating back to December 24, 2019 (EPI_ISL_402123).

SARS-CoV-2 genomes were aligned to the 29,903 nt-long reference assembly of SARS-CoV-2 by means of the *nucmer* program (Marçais et al. 2018). Custom Perl scripts were used to infer the size of each genomic assembly and the number of uncalled bases/gaps (denoted by N in the genomic sequence). Only genome sequences longer than 29,850 nt and including less than 150 ambiguous sites were analyzed.

Variant sites, including substitutions and small insertion and deletions, were identified by using the *show-snps* utility of the *nucmer* package. Output files were processed by the means of a custom Perl script, and converted into a phenetic matrix, with variable positions on the rows and viral isolates in the columns. Values of 1 and 0 were respectively used to indicate presence or absence of a variant.

Genetic distances between genomic sequences were established from this phenetic matrix using the *dist* function of the R *stat* package with default parameters (Euclidean distances) (Maechler et al. 2019; R Core Team 2019). Clusters were established by means of hierarchical clustering algorithms, with complete linkage as implemented in the *hclust* R standard libraries function. The *cutree* function was used to separate distinct clusters at the desired level of divergence (two distinct variant sites). Functional effects of genetic variants, as identified from genome alignments, were predicted by means of a custom Perl script, based on the annotation of the NC_045512.2 SARS-CoV-2 reference assembly.

Alignments of SARS-CoV-2 protein-coding genes were performed by the means of the Muscle (Edgar 2004) software. Alignments were concatenated using a custom Perl utility. The SMS (Smart Model Selection) algorithm, as implemented by the PhyML package (Lefort et al. 2017) was used for the selection of the most appropriate aminoacid substitution model. The WAG (Whelan and Goldman 2001) model was selected. A phylogeny was reconstructed using the FastTree program (Price et al. 2009). Identification of sites under selection was performed by applying the MEME and FEL methods, as implemented in the *Hyphy* package (Kosakovsky-Pond et al. 2020), to the phylogeny and the concatenated alignment of protein-coding sequences of all the 102,951 previously identified high-quality complete SARS-CoV-2 genomes. A *P* value of 0.05 was considered for the significance threshold. Only sites predicted to be under positive selection according to both methods were considered. For sites predicted to be under negative selection, only FEL was used, since MEME can not identify purifying selection (Murrell et al. 2012).

A total of 68 viral genomes from the SARS 2003 outbreak were retrieved from the NCBI virus database (Goodacre et al. 2018). Calculation of evolutionary rates of SARS-CoV-2 and estimation of times of divergence were performed according to the formula described in Zhao et al. 2004, based on genetic distances as determined in this study.

Analyses of prevalence of allele frequency over time were executed based on the collection dates of individual genomes as reported in the GISAID metadata table. The collection date of the reference genomic sequence of SARS-CoV-2 in GISAID (December 26, 2020), was set as time T0. Consecutive, nonoverlapped intervals of 10 days were considered. For the analysis of allele frequency within haplogroups and major haplogroups, the cumulative (from T0) sequence distribution was computed at every interval. Global analyses of allele prevalence within the complete collection of SARS-CoV-2 genomes were executed on the equivalent time intervals of 10 days, in this case however to capture local effects in allele frequency variation, overlapped (by 5 days) intervals were considered and the local distribution of allele frequency was computed by taking into account only the genomes isolated within each specific interval of time.

A total of 3608 genomic sequences, for which collection dates were not reported in GISAID, were excluded from these analyses.

Comparison of levels of variability of “early” and “late” clusters of SARS-CoV-2 genomes were established by 100 random samples of 150 genomes (batch), matched by date of collection, from each defined cluster (see below). The total number of distinct variant sites was calculated for each random batch of genomes, in order to derive a distribution of genomic variability. Significance between distributions of genomic diversity was established by means of the Wilcoxon, sum, and rank test as implemented in the standard R libraries (R Core Team 2019).

Variability with respect to the reference NC_045512.2 SARS-CoV-2 genome was computed on sliding windows of 100 bp, overlapped by 50 bp, by counting the proportion of variable sites contained in each window (number of variable sites in the window, divided by the total number of variable sites in the entire genome) with a custom Perl script. A Fisher-exact test, contrasting the local variability in a window with the average variability in the genome, was used to identify hypervariable regions. *P* values were corrected using the Benjamini–Hochberg procedure for the control of false discovery rate.

Predictions of the secondary structure of the “Coronavirus stem-loop II-like motif” (s2m) and its Minimum Folding Energy (MFE) calculation were performed with the RNAfold program (Mathews et al. 2004) of the Vienna package (Gruber et al. 2008), by artificially implanting each of the possible 129 substitutions in the 43 nt-long s2m sequence identified in the reference SARS-CoV-2 genome and in the presumably ancestral sequence of s2m, that is, that observed in the genome of the RatG13 SARSr-CoV-2.

Prediction of the consensus cofolding structure of s2m in SARS-CoV-2 was obtained by applying the R-scape (Rivas et al. 2017) program to the alignment of all s2m sequences found in the collection of the 11,633 high-quality complete genome analyzed in this study.

Consensus secondary structure of the s2m element of Coronaviruses was as in the model RF00164 (https://rfam.org/family/RF00164) of the RFAM database.

Graphical representation of the data, basic statistical analyses, and clustering of viral genomes were performed by means of the standard libraries of the R programming language.

A software package for the assignment of SARS-CoV-2 haplogroups as proposed in this work is made publicly available through this Github repository https://github.com/matteo14c/assign_CL_SARS-CoV-2 and also in the form of a public galaxy server with a collection of tools for the annotation of SARS-CoV-2 genomes at http://corgat.cloud.ba.infn.it/galaxy.

## Supplementary Material

Supplementary data are available at *Molecular Biology and Evolution* online.

## Supplementary Material

msab049_Supplementary_Data
