# Supplementary material for: Comparative Genomics Reveals Early Emergence and Biased Spatiotemporal Distribution of SARS-CoV-2
Source: Mol Biol Evol. 2021 Feb 19;38(6):2547–65. doi: 10.1093/molbev/msab049 (PMC7928790; doi:10.1093/molbev/msab049)

List of Supplementary Materials, and corresponding legends

Supplementary Figures:

Supplementary Figure 1. Temporal persistence of high frequency alleles
A) Persistence, in days of high frequency (>1%) variants. The barplot represents the total number of variants that reach a high frequency (>1%) for different time intervals in the genome of SARS-CoV-2. Number of variants is reported on the Y axis, days are indicated on the X axis.
B) Correlation between maximum allele frequency and persistence (AF>1%) of genetic variants. Maximum allele frequency is represented on the X axis, time of persistence (allele frequency>1%) in days is reported on the Y axis.

Supplementary Figure S2: Clustering of 178,191 SARS-CoV-2 genomes.
A) Heatmap of presence/absence of 82 high frequency polymorphic sites (AF >0.01 for more than 50 days) in the complete collection of 178,191 SARS-CoV-2 genomes, assigned to the 22 haplogroups here identified. Genomic coordinates are represented on the X axis. Light gray indicates a reference allele, dark gray an alternative allele for that site. The panels on the left indicate haplogroups, with a different colour assigned to each. Dotted lines delineate macrohaplogroups.
B) Bubbleplot of allele frequency of the 82 high frequency polymorphic sites in each haplogroup. Color codes according to Figure 1A. The dendrogram on the left indicates haplogroups with similar allele frequency profiles. The size of each “bubble” is proportional to the frequency of that allele in a given haplogroup. Barplot on the panel indicates the number of genomes assigned to every haplogroup, scaled by logarithm base 10.

Supplementary Figure S3: Prevalence, in time of genetic variants specific to MHG3. A) 241 C>T. B) 3037 C>T. C) 14408 C>T. D) 23403 A>G. Prevalence is indicated on the Y axis, time intervals are indicate on the x axis

Supplementary Figure S4: Prevalence of different haplogroups of SARS-CoV-2, at different intervals of time in A) Australia, B) USA and C) UK. Color codes above indicate individual haplogroups, according to Figure 1. Time points with respect to the date of isolation of the reference genome, are indicated on the right. Barplots are used to show the (log10) total number of genomic sequences associated/available at every time point.

Supplementary Figure S5: Prevalence of haplogroups and macrohaplogroups in different geographic regions of China
A) Prevalence of macrohaplogroups and haplogroups in four distinct regions of China, for which more than 20 genomic sequences were collected within 25 days of the collection date of the reference genome (26th December 2019). Pie-charts represent the prevalence of haplogroups. Outer circles indicate the prevalence of macro haplogroups.
B) Prevalence of macrohaplogroups and haplogroups in Wuhan city within 25 days of the collection date of the reference genome (26th December 2019). Pie chart as described above. In the heatmap genomic coordinates are represented on the X axis. Pink indicates a reference allele, red an alternative allele for. Panels on the left indicate haplogroups, with a different colour assigned to each. Color codes according to Figure 1. Collection dates are reported on the rows.

Supplementary Figure S6: Phenetic patterns of the 82 high frequency SARS-CoV-2 alleles, in SARSr-CoVs-2. SARS-CoV-2 genomic positions for the 82 high frequency (AF>=0.01) polymorphic sites of SARS-CoV-2 are represented on the X axis. Color codes are used to indicate alleles that are prevalent in different MHGs. Light colors are used to indicate variants that are fixed in a macrohaplogroup. Darker colors indicate variants that are fixed only in a limited number of

haplogroups. GISAID accession numbers and host species from which the genomes were isolated (pangolin or bat) are reported on the rows.

Supplementary Figure S7: Bubbleplot of allele frequency MHG2. Allele frequency of the 82 high frequency polymorphic sites in haplogroups included in MHG2, calculated at different and non-overlapping intervals of 10 days (“T_”, with time 0 = 26th December 2019, i.e., the collection date of the reference genome). The barplots above indicate the number of genomes at each time interval considered.

Supplementary Figure S8: Bubbleplot of allele frequency MHG3. Allele frequency of the 82 high frequency polymorphic sites in haplogroups included in MHG3, calculated at different and non-overlapping intervals of 10 days (“T_”, with time 0 = 26th December 2019, i.e., the collection date of the reference genome). The barplots above indicate the number of genomes at each time interval considered..

Supplementary Figure S9: Bubbleplot of allele frequency MHG4. Allele frequency of the 82 high frequency polymorphic sites in haplogroups included in MHG4, calculated at different and non-overlapping intervals of 10 days (“T_”, with time 0 = 26th December 2019, i.e., the collection date of the reference genome). The barplots above indicate the number of genomes at each time interval considered.

Supplementary Figure S10: Prevalence, in time of genetic variants in the spike protein specific to HG18/HG21 and HG15. A) L18F (HG21). B) A222V (both HG18 and HG21). C) S477N (HG15). Prevalence is indicated on the Y axis, time intervals are indicated on the X axis

Supplementary Figure S11. Time of emergence of first 50 isolated genomes in each of the 22 SARS-CoV-2 haplogroups. Violin plots of isolation dates of SARS-CoV-2 strains assigned to each haplogroup of SARS-CoV-2 genomes. Color codes according to Figure 1. Haplogroups are indicated on the Y axis. Isolation dates are reported on the X axis.

Supplementary Figure S12: Phenetic patterns and geographic area of the first 82 genomes for each “early” haplogroups. Each heatmap displays presence/absence of the 82 high frequency polymorphic sites (AF >0.01, identified in the 102,951 “high quality” complete SARS-CoV-2 genomes) in the first 50 genomes, in terms of date, of each early haplogroups. Genomic coordinates are represented on the X axis. Light gray indicates a reference allele, dark gray an alternative allele for that site. Panels on the left indicate continents: yellow=Asia, blue=Europe, pink=Africa, green=North America, orange=South America, purple=Oceania. Country and date of collection are reported on the rows.

Supplementary Figure S13: Phenetic patterns and geographic area of the first 82 genomes for each “middle” haplogroups. Each heatmap displays presence/absence of the 82 high frequency polymorphic sites (AF >0.01, identified in the 102,951 “high quality” complete SARS-CoV-2 genomes) in the first 50 genomes, in terms of date, of each early haplogroups. Genomic coordinates are represented on the X axis. Light gray indicates a reference allele, dark gray an alternative allele for that site. Panels on the left indicate continents: yellow=Asia, blue=Europe, pink=Africa, green=North America, orange=South America, purple=Oceania. Country and date of collection are reported on the rows.

Supplementary Figure S14: Phenetic patterns and geographic area of the first 82 genomes for each “middle” haplogroups. Each heatmap displays presence/absence of the 82 high frequency polymorphic sites (AF >0.01, identified in the 102,951 “high quality” complete SARS-CoV-2 genomes) in the first 50 genomes, in terms of date, of each early haplogroups. Genomic coordinates

are represented on the X axis. Light gray indicates a reference allele, dark gray an alternative allele for that site. Panels on the left indicate continents: yellow=Asia, blue=Europe, pink=Africa, green=North America, orange=South America, purple=Oceania. Country and date of collection are reported on the rows.

Supplementary Figure S15: Plot of genome-wide genetic variability of SARS-CoV-2 haplogroups. Plot of genomic variability, calculated as the proportion of variable sites identified in overlapping genomic windows of 100 bp in the 22 haplogroups HG1-HG22. Genomic coordinates are represented on the X axis, number of variable sites per window on the Y axis. Color codes according to Figure 1

Supplementary Tables:

Table S1. Collection of genomes used in our study. Isolate name and identifier according to the GISAID database are reported in the first column. Days (from the day of the collection of the first genome, 26th December) and date of isolation are reported in column 3 and 4 respectively. Geographic metadata, including continent, country, region and city are reported in columns 5 to 8. Minimal clinical metadata: host, age and sex are indicated in columns 9 and 10. Finally classification of genomes, according to Nextstrain, Rambaut et al. (2020) (pangolin) GISAID, and our novel method are reported in columns 11 to 14. The last three columns are used to indicate, genomes that contain a high number (>250) of ambiguous bases (0= FALSE, 1=TRUE), genomes providing incomplete representation of the UTR regions (>250 bp missing, 0= FALSE, 1=TRUE) and genomes that are complete and of high quality according to our criteria (0= FALSE, 1=TRUE). Only the latter (high quality genomes) have been included in our analyses.

Table S2. Functional annotation of SARS-CoV-2 genetic variants. Functional annotation of the 28,222 genetic variants observed in genomes of SARS-CoV-2 . Columns 1 to 3 report: 1) genomic coordinates, 2) and 3) reference and alternative allele. Functional annotation is reported in column 4. Columns 5 to 7 are used to indicate 5) genetic variants that reach a frequency above 1%, 6) variants that show a prevalence of above 1% for more than 50 days in total (0 false, 1 true) and 7) variants that are completely fixed in one HG (> 90% of isolates). (0= FALSE, 1=TRUE)

Table S3. Prevalence of SARS-CoV-2 genetic variants over time. Columns 1 to 3 report genomic coordinates (1), reference (2) and alternative (3) allele. Column 4 indicates the total number of days in which variants show an allele frequency of 1% or above. Prevalence at different time intervals is reported in the subsequent columns.

Table S4. Prevalence of Genetic variants within the 22 SARS-CoV-2 haplogroups. A) List of the variants that are completely fixed (> 90% frequency) in one haplogroup. B) prevalence of the 82 variants that reach complete fixation in at least one haplogroup, in all the 22 SARS-CoV-2 haplogroups

Table S5. List of variants under positive or negative selection as inferred by MEME and FEL methods A) Positive selection. B) negative selection.

Table S6. Allele frequency of the 82 high frequency polymorphic sites in the 4 macro-haplogroups of genomes defined in this study. "T_" indicates time expressed in days, with time 0= 26th December 2019, i.e., the collection date of the reference genome

Table S7. Allele frequency of the 82 high frequency polymorphic sites in the 22 haplogroups of genomes defined in this study. "T_" indicates time expressed in days, with time 0= 26th December 2019, i.e., the collection date of the reference genome

Table S8. Analysis of stability of secondary structure of the s2m element. Rel pos: relative position in s2m. Gen pos: genomic position. Sub: Nucleotide substitution. MFE: MFE. Observed: Observed in an actual genome sequence: 1=True, 0=False. Sub_RaTG13: Nucleotide substitution in the s2m of the RaTG13 genome. MFE_RaTG13: MFE in the s2m of the RaTG13 genome. The first row indicates the MFE (minimum free energy) of s2m secondary structure found in the reference genome of SARS-CoV-2 and in the RaTG13 SARSr-CoV-2 genome assembly. NA=not applicable.

Supplementary Figure S1

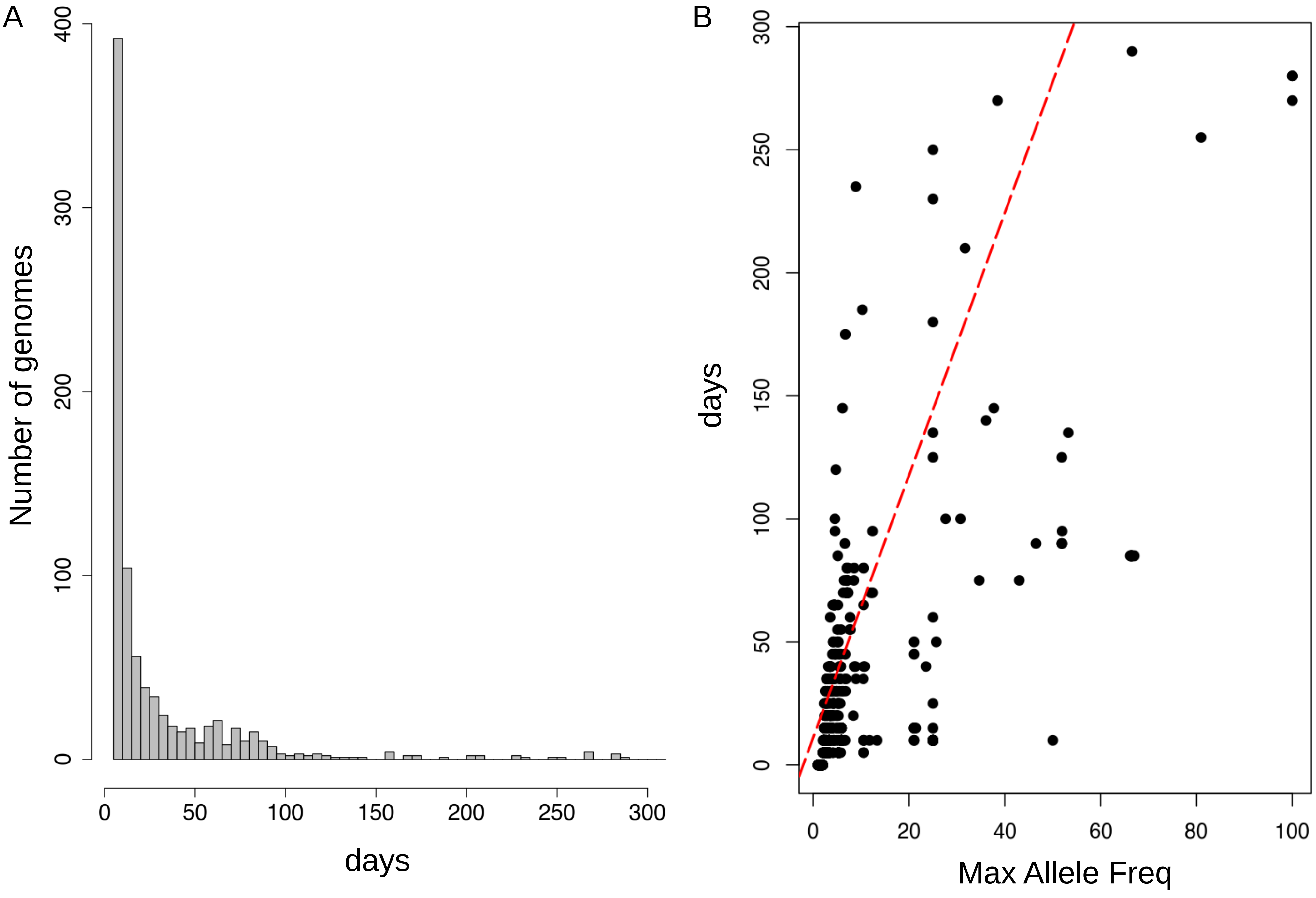

Supplementary Figure S2

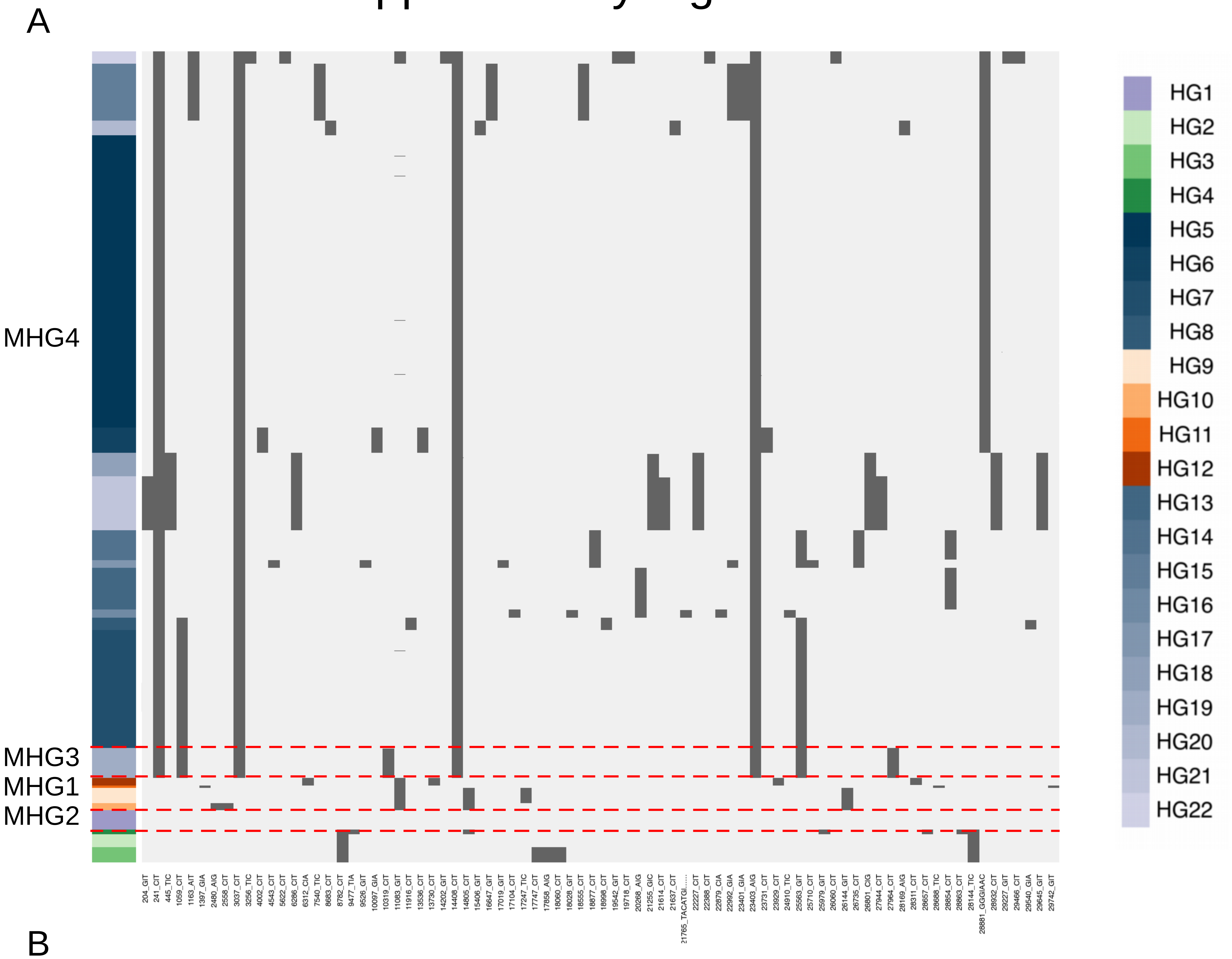

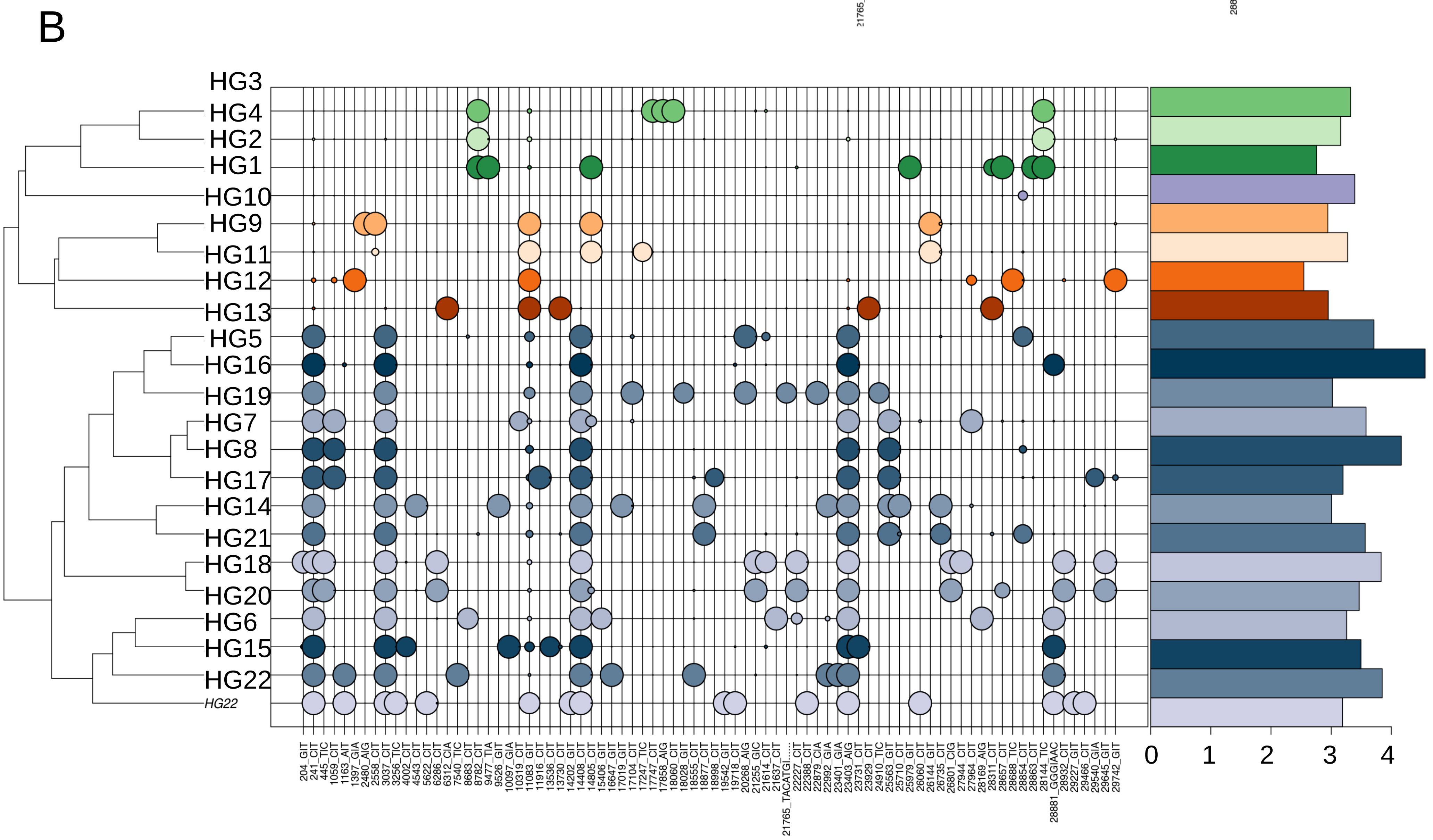

Supplementary Figure S3

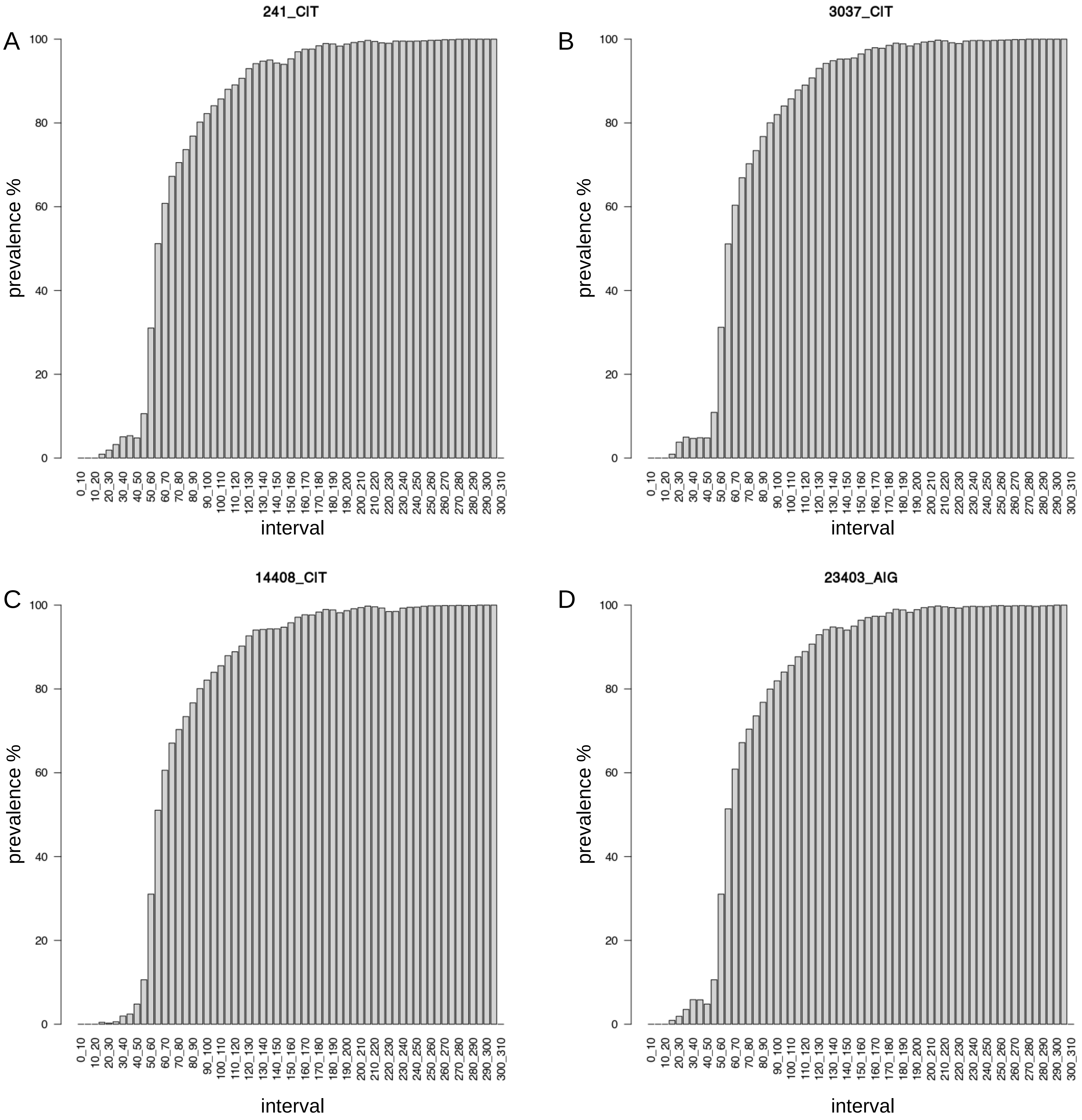

Supplementary Figure S4

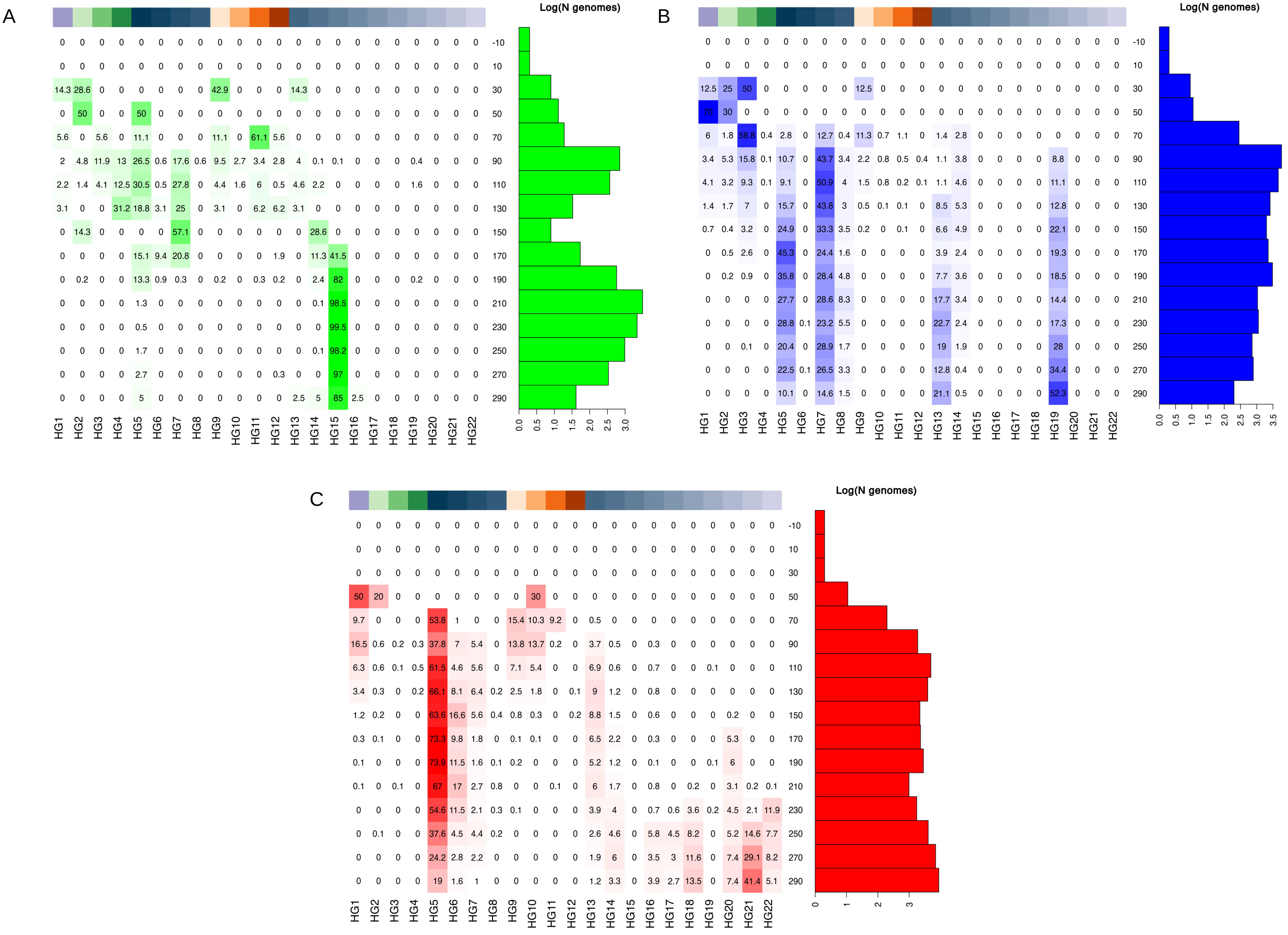

Supplementary Figure S5

A

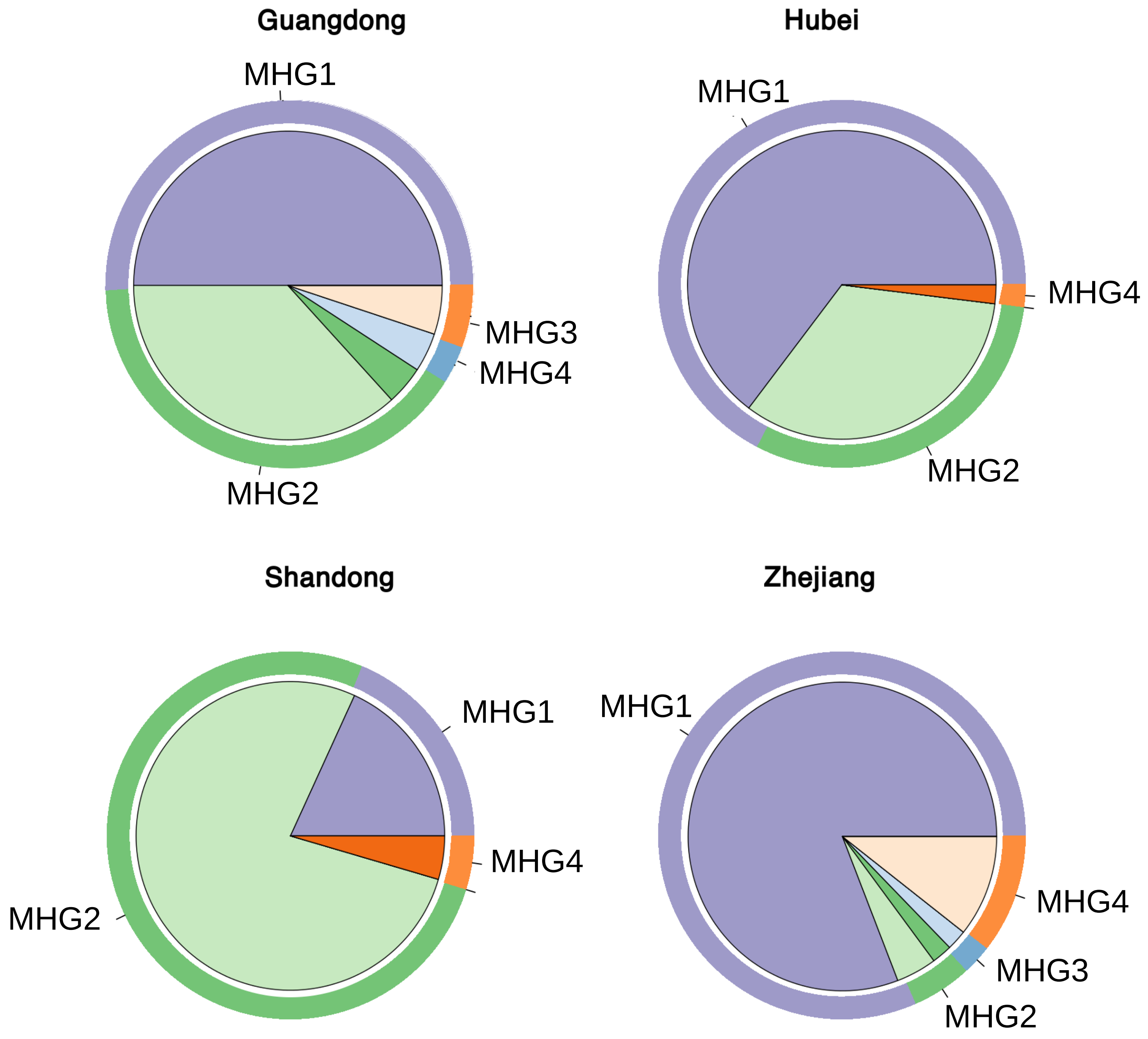

B

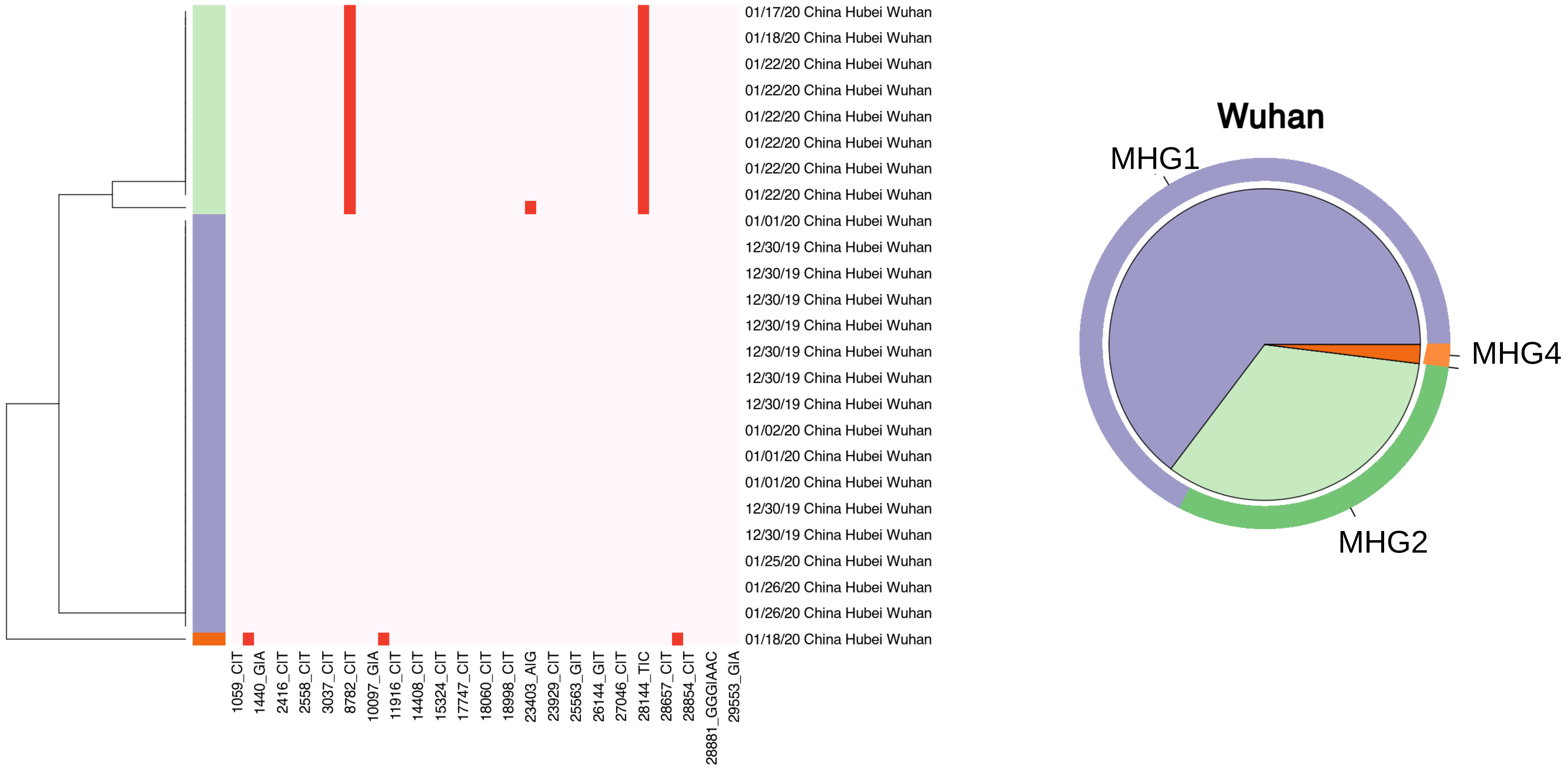

Supplementary Figure S6

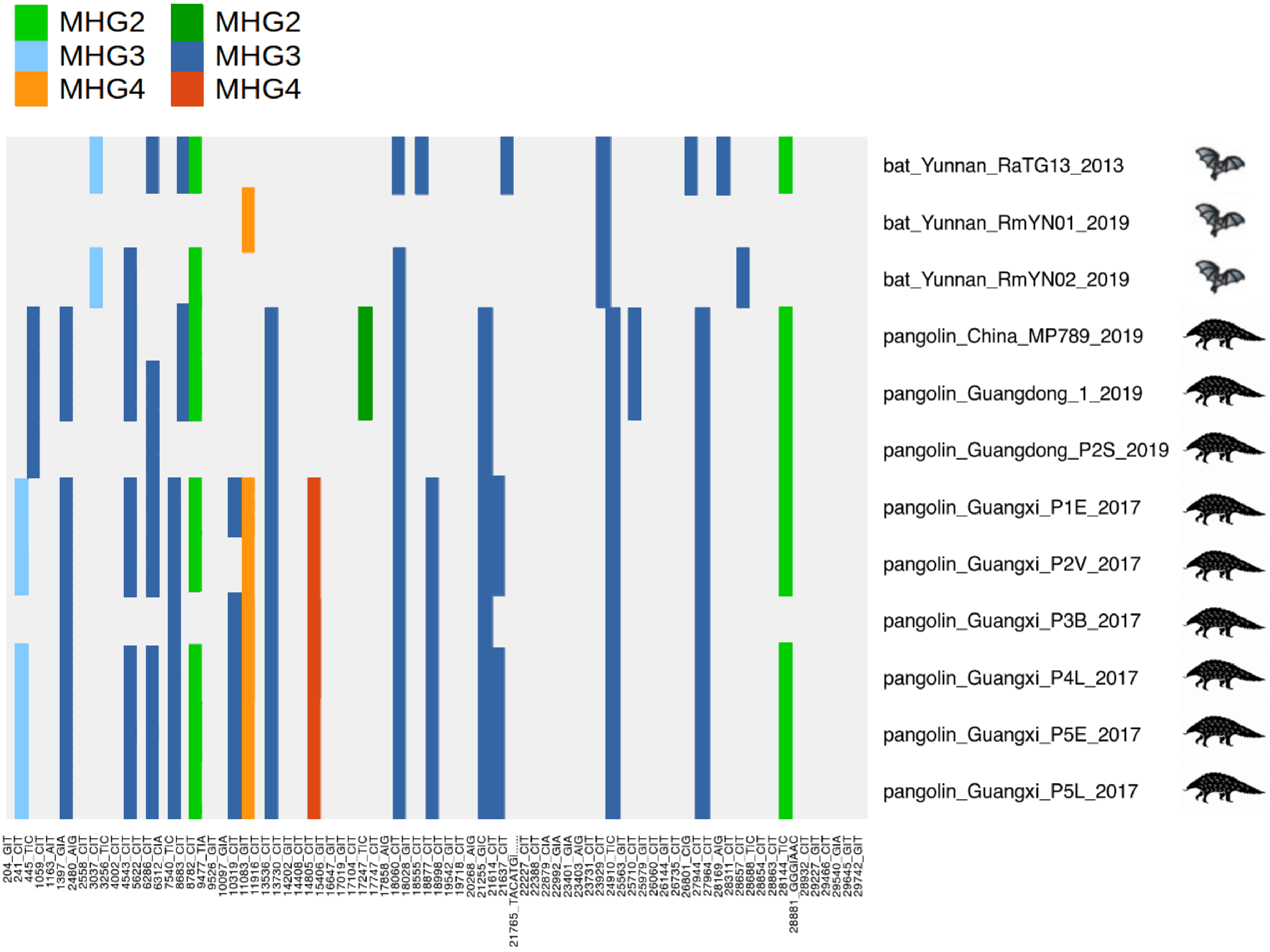

Supplementary Figure S7

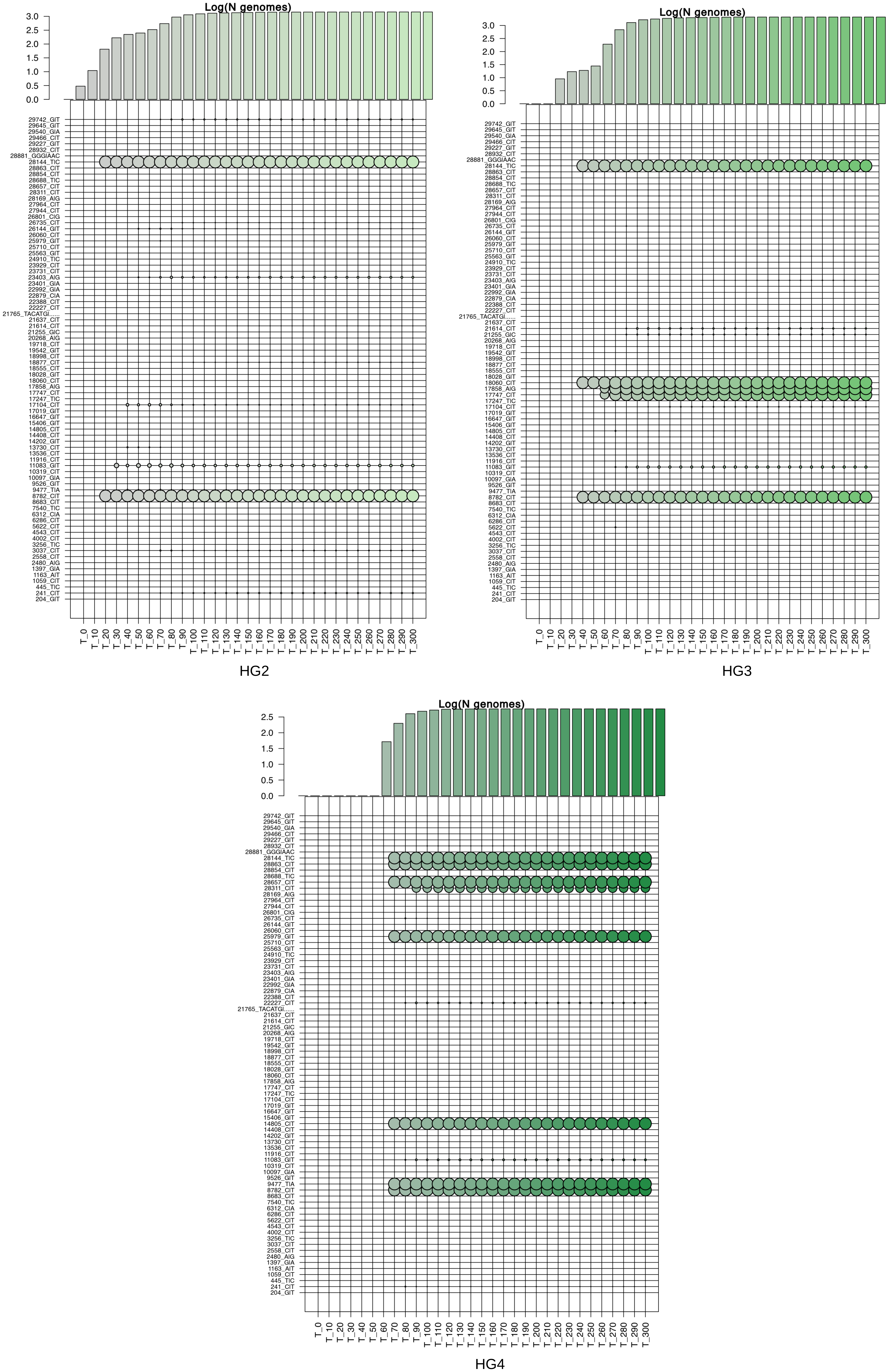

Supplementary Figure S8

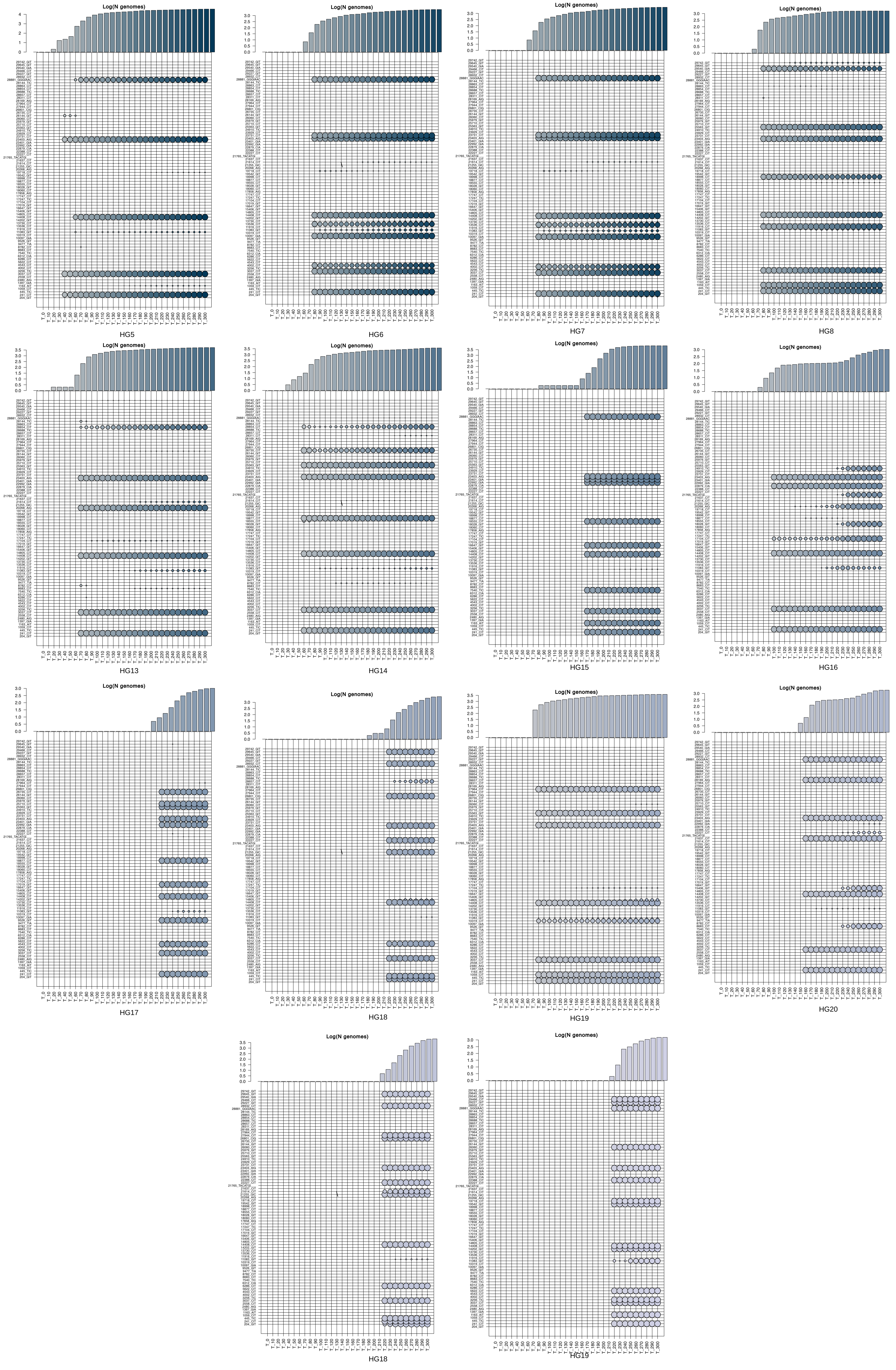

Supplementary Figure S9

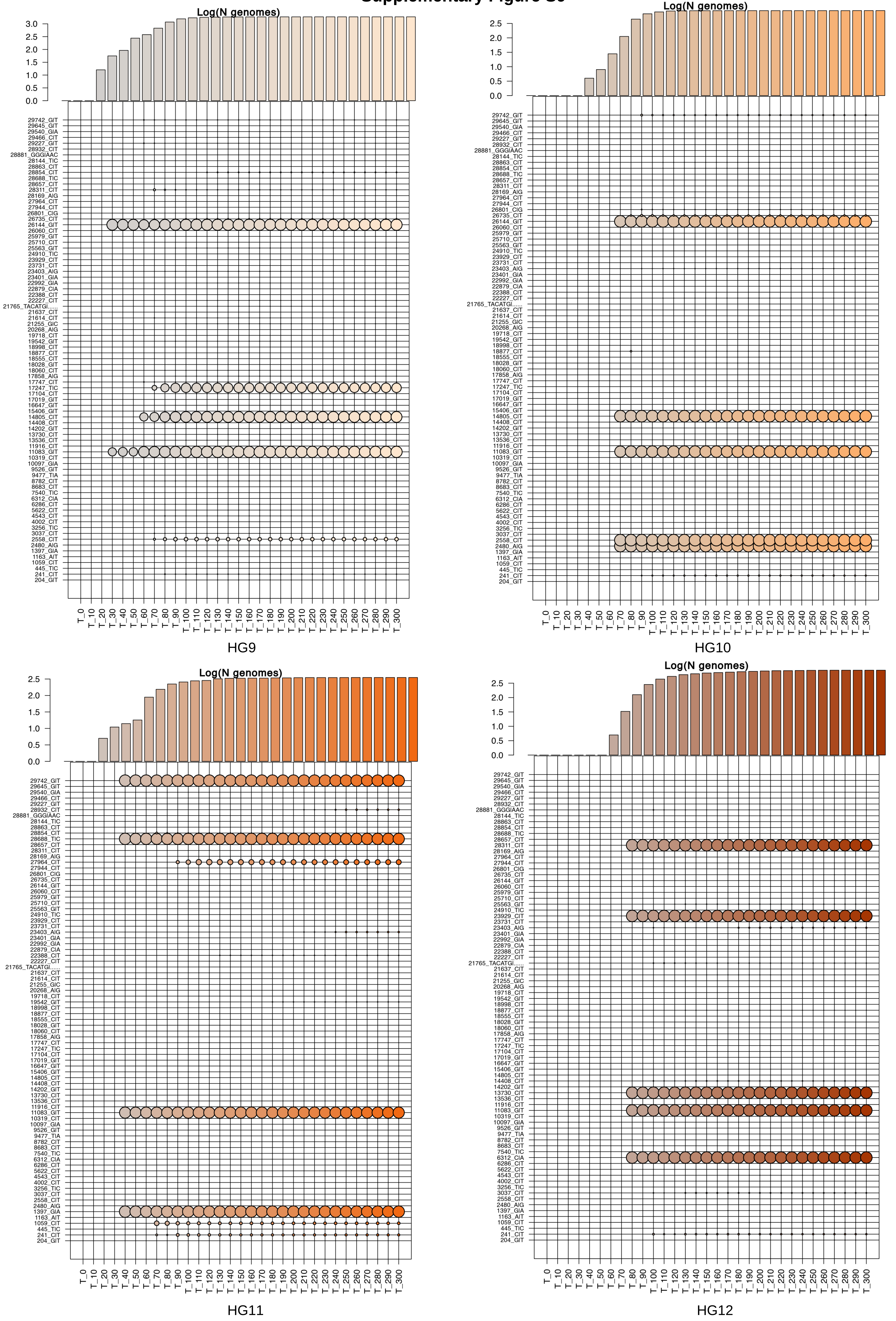

Supplementary Figure S10

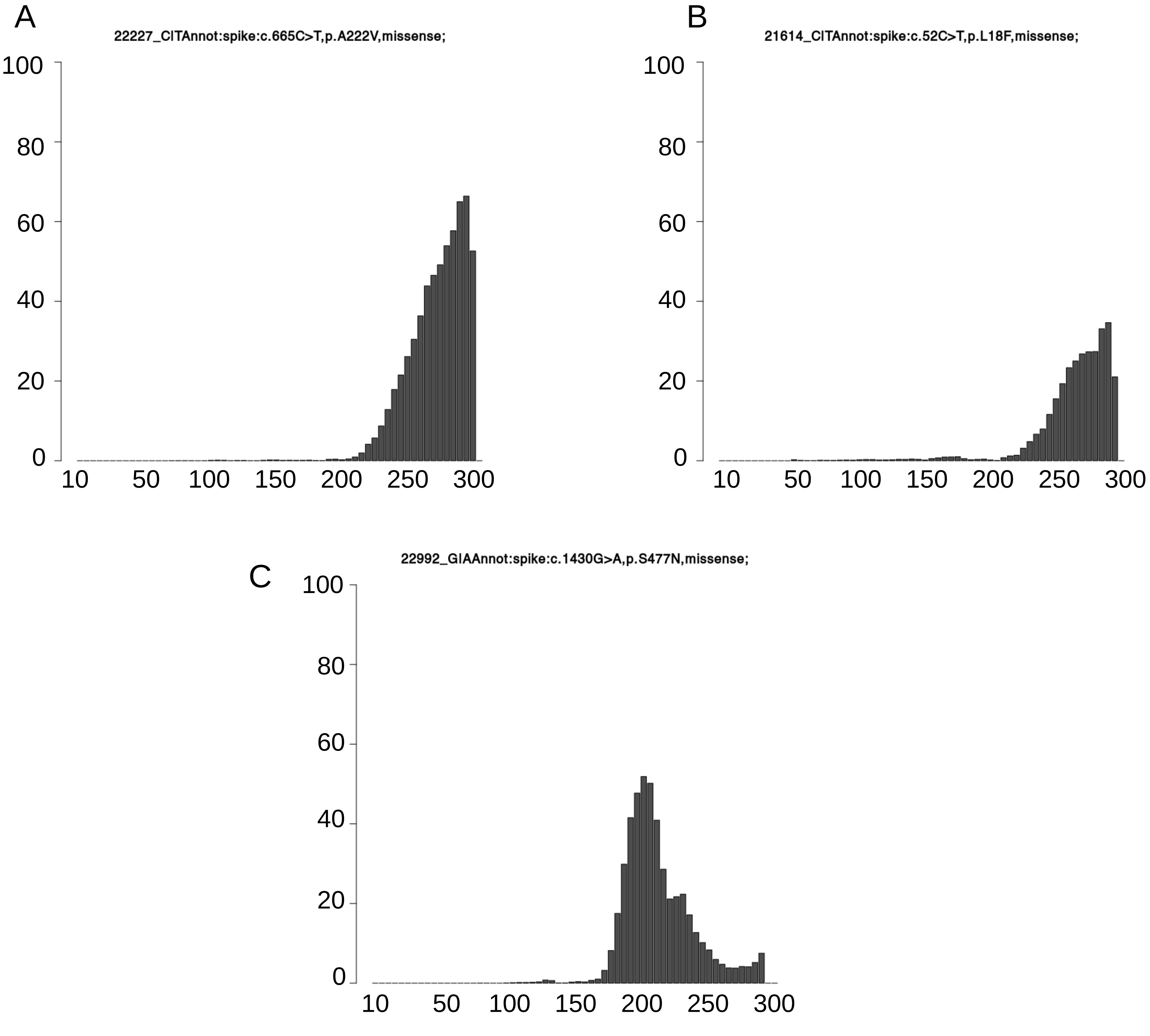

Supplementary Figure S11

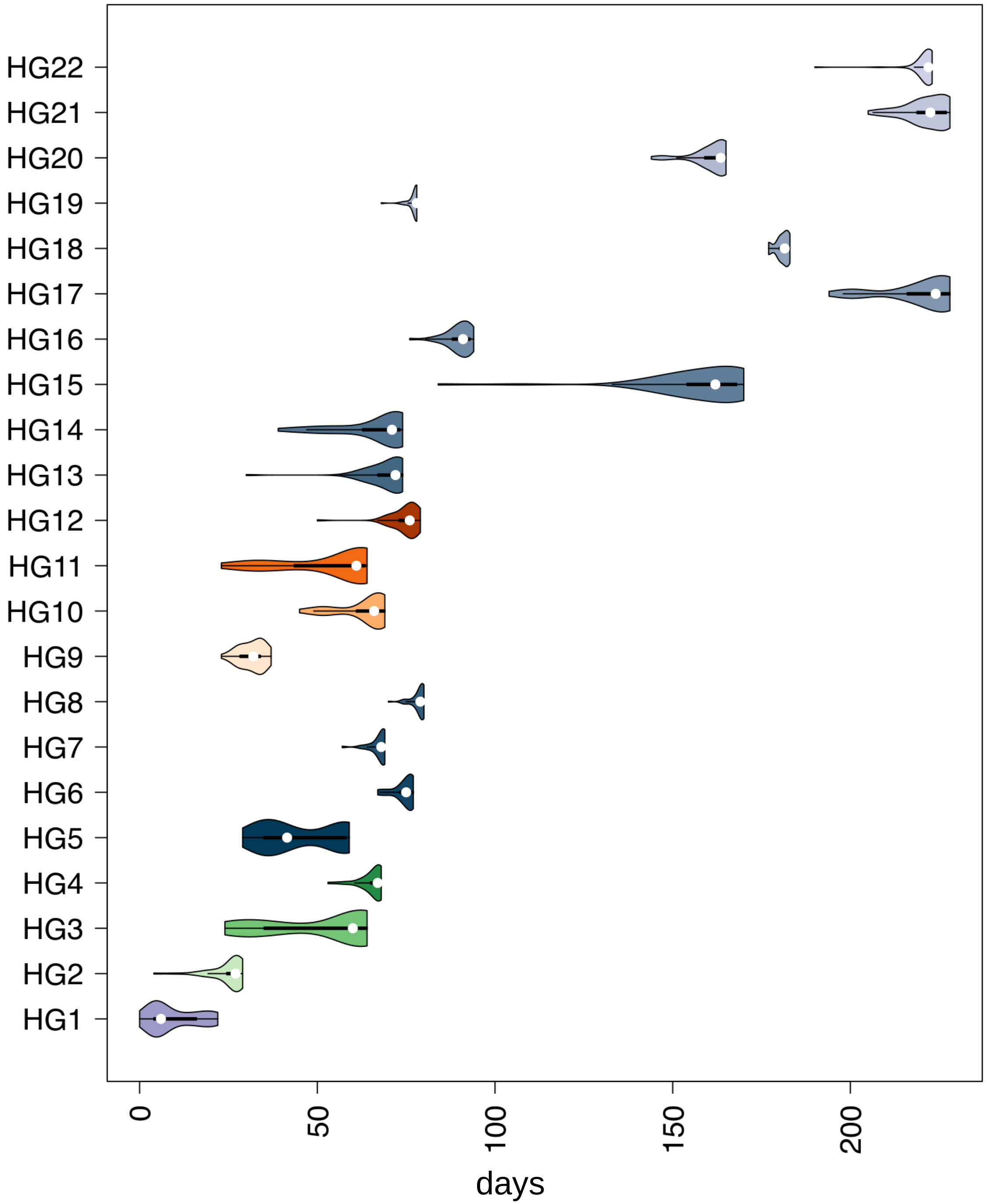

Supplementary Figure S12

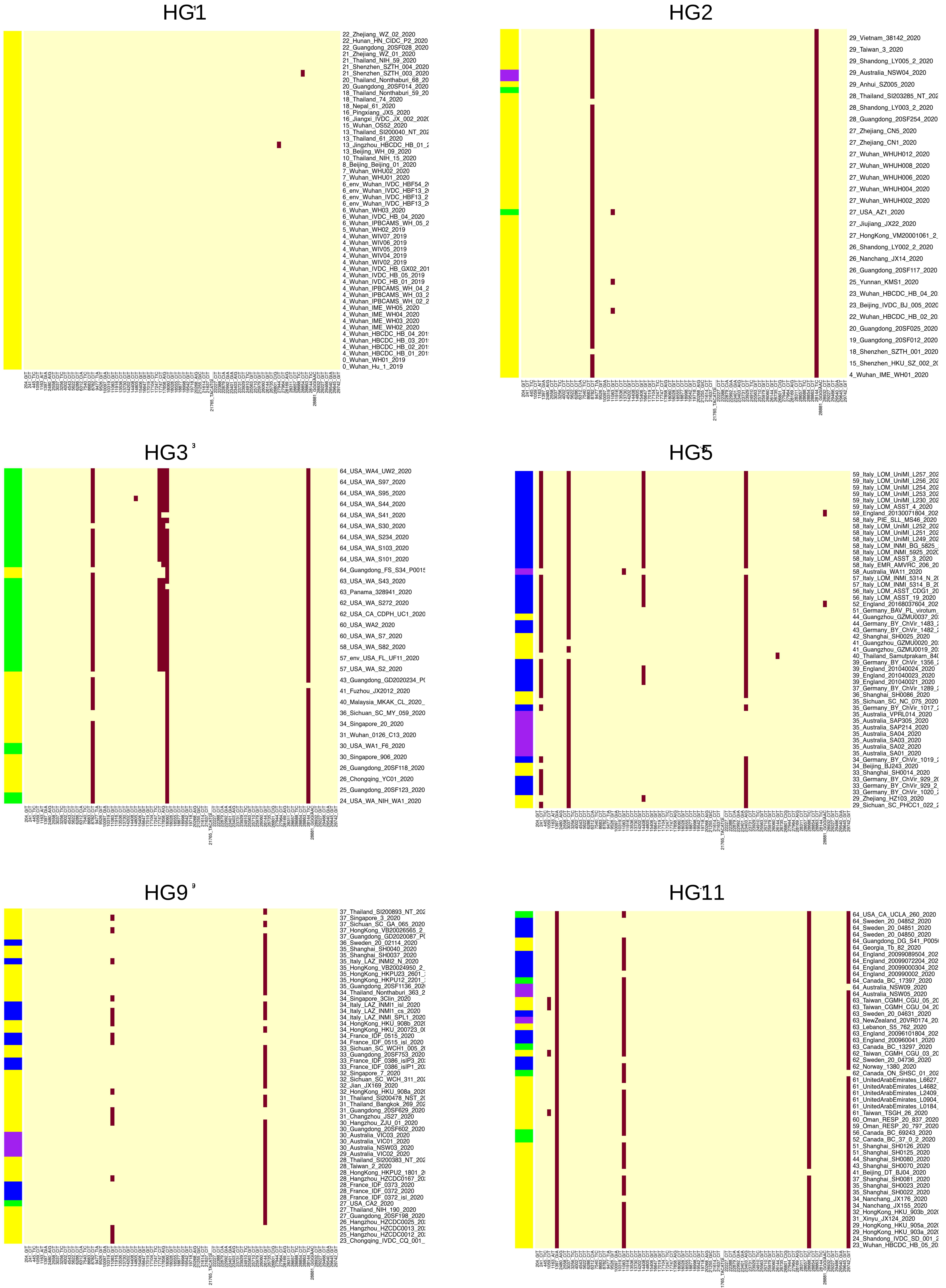

Supplementary Figure S13

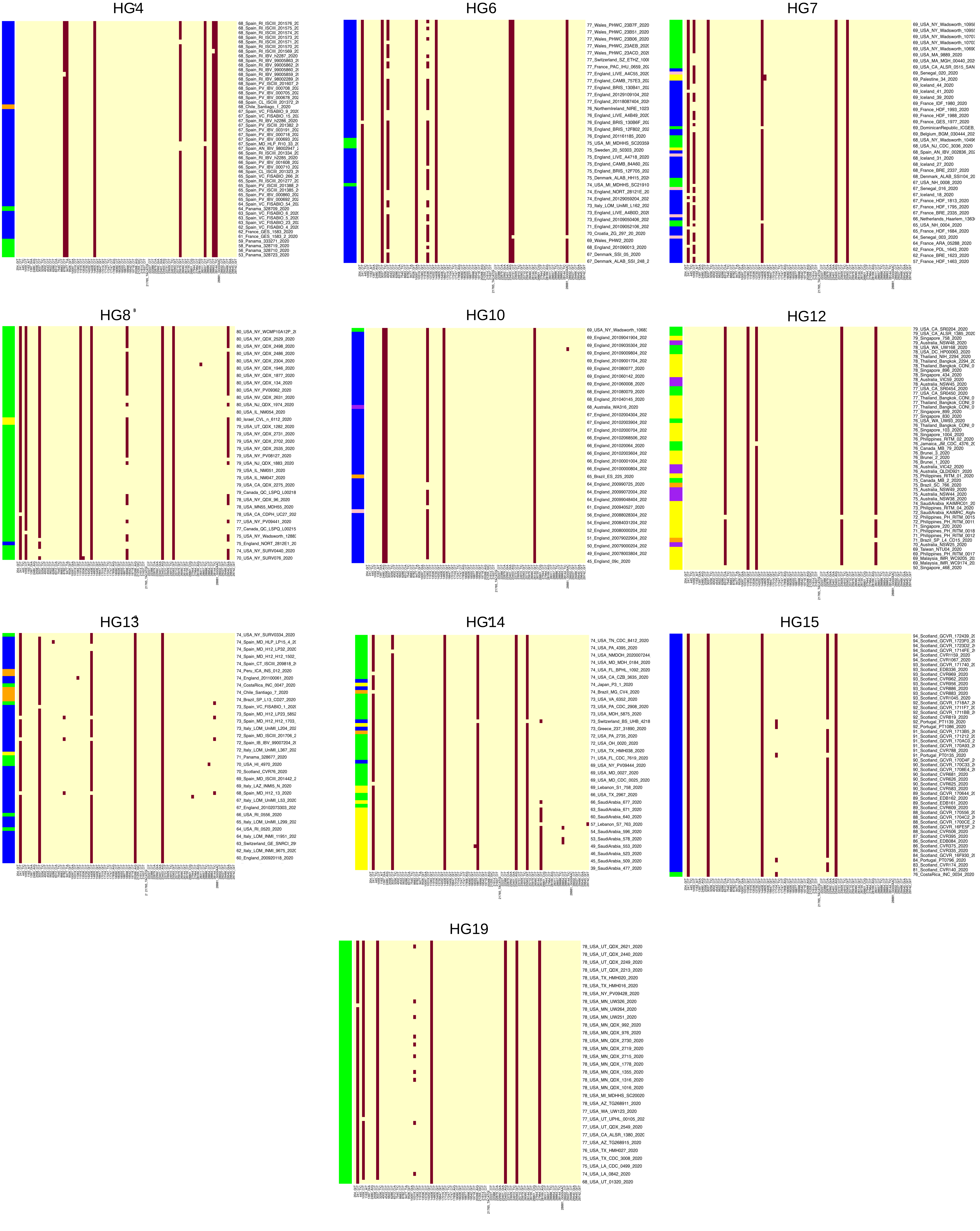

Supplementary Figure S14

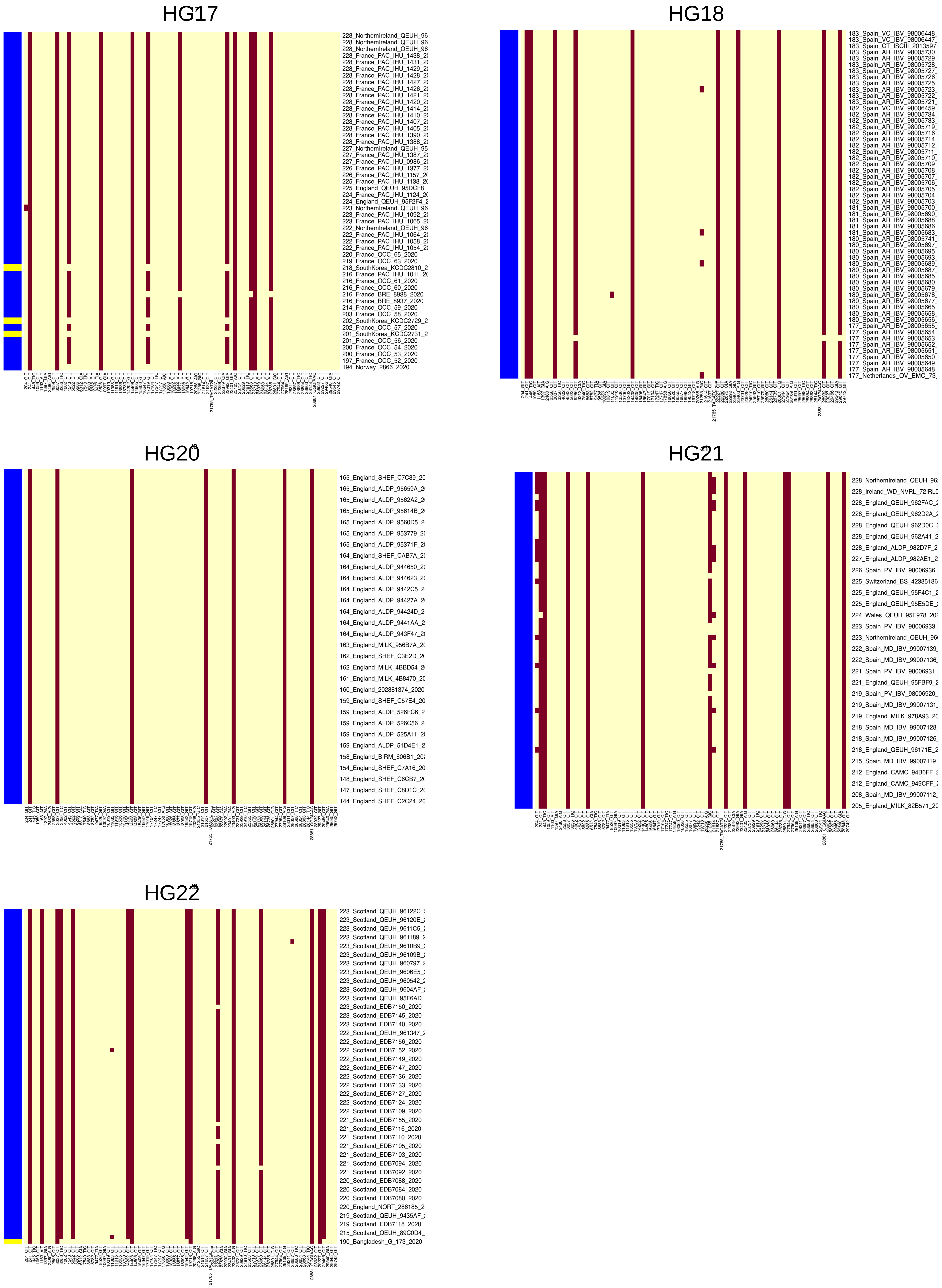

Supplementary Figure S15

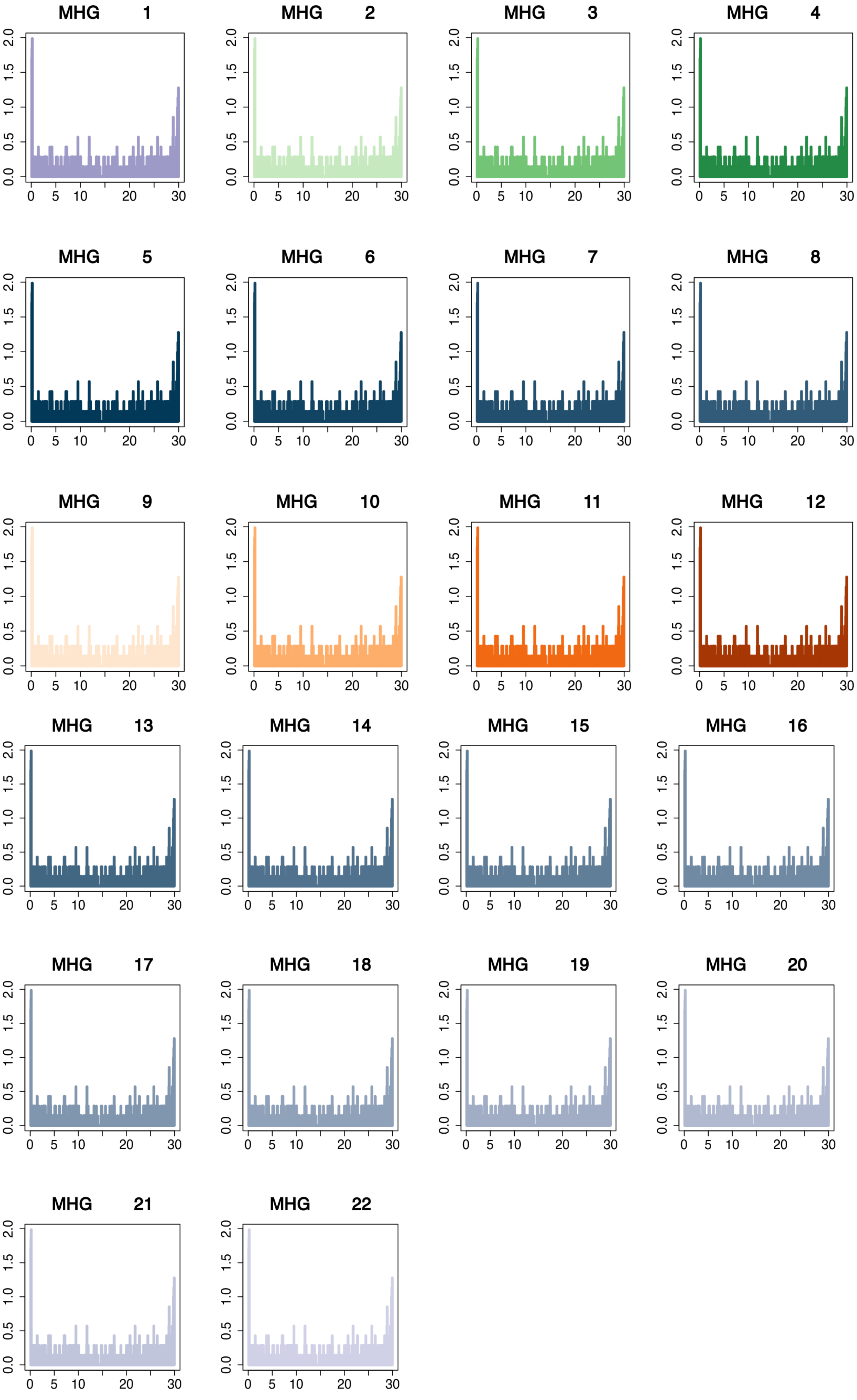

Supplement: msab049_Supplementary_Data [file msab049_supplementary_data.zip › MBE_production_complete_supplemetaryFigures_plus_legends.pdf]
